# Sample size determination across study designs in health professions education research: a methodology guide

**DOI:** 10.12688/f1000research.185853.1

**Published:** 2026-07-13

**Authors:** Waqar M Naqvi, Anusha Divvi, Shivashankar Kengadaran, Aishwarya A Pashine, Maheshkumar Baladaniya, Aditya Patel, Praveen Kumar Kandakurthi, Ramprasad Muthukrishnan

**Affiliations:** 1 Department of Health Professions Education, Faculty of Interdisciplinary Sciences, Datta Meghe Institute of Higher Education and Research, Wardha, India; 2 Public Health Dentistry, Indira Gandhi Institute of Dental Sciences, Sri Balaji Vidyapeeth University, Puducherry, Puducherry, India; 3 Department of Technical Medicine (Medical Simulation), Faculty of Interdisciplinary Sciences, Datta Meghe Institute of Higher Education and Research, Wardha, India; 4 Department of Physical Therapy, Neighborhood Physical Therapy PC, Newyork, USA; 5 Department of Conservative Dentistry and Endodontics, Sharad Pawar Dental College and Hospital, Datta Meghe Institute of Higher Education and Research, Wardha, India; 6 College of Health Sciences, Gulf Medical University, Ajman, Ajman, United Arab Emirates

**Keywords:** Sample size, Sample size calculation, Statistical power, Effect size, Research design, Health professions education

## Abstract

**Background:**

Sample size determination is one of the least reported elements in the methods sections of health professions education (HPE) research. Underpowered studies miss real effects, whereas overpowered studies waste participants and resources. Guidance on sample size remains fragmented, distributed across statistical textbooks and discipline-specific papers, with no unified reference for HPE researchers.

**Methods:**

Rather than proposing a universal formula, which does not exist, we organised sample-size logic by study design, reflecting how investigators plan their research. We set out a stepwise determination workflow and emphasise sourcing the assumed effect size from a pilot study or a comparable parent study. For each design we present the statistical rationale, the applicable formula or rule of thumb, a worked example grounded in HPE practice, and explicit reporting expectations.

**Results:**

The designs covered include two-group comparisons of means and proportions, pre–post and repeated-measures designs, cluster-randomized educational trials, survey and questionnaire studies, Delphi and consensus studies, pilot and feasibility studies, qualitative enquiry, simulation-based education, and instrument validation. The guide concludes with reporting standards for the methods section, written to serve investigators, peer reviewers, and journal editors.

**Conclusions:**

Sample size in HPE research is a design-specific judgment, not a formula-specific calculation. This guide consolidates that judgment into a practical and rigorous reference that reflects the methodological diversity of the field.

## Introduction

Sample size is one of the most consequential design decisions in any health professions education (HPE) study, yet it is routinely under-reported and under-justified. A systematic review of sample size adequacy in HPE research reported a median sample size of 25 per study and found that fewer than 1% had ≥80% statistical power to detect a small standardized effect (SMD > 0.2), while only about one in five could detect a large effect (SMD > 0.8). ^1^ This suggests that, under realistic effect sizes for educational interventions, a substantial proportion of the existing HPE evidence base lacks adequate statistical power. Underpowered studies have a low probability of detecting true effects, but they also have a low positive predictive value.

Sample size errors in HPE research fall into recognizable patterns. Reviews of methodological quality in medical and health professions education have documented that sample-size justifications are frequently absent, are restricted to a single number with no stated effect size or assumptions, or rely on rules of thumb that do not match the analytical model being used. ^2^ ^,^ ^3^ A particularly common error in cluster-delivered interventions is to treat learners within classrooms, programs, or clinical sites as independent observations, ignoring the intracluster correlation and overstating the effective sample size. ^4^ A different but equally common error is post hoc justification, calculating or describing power after data have been collected, a practice that is uninformative for inference, and the argument provides no useful information beyond the p-value itself. ^5^ An earlier review by some of the present authors documented variability in sample size practices in published educational research. ^6^ The current guide provides the design-driven methodological grounding needed to address that variability.

There is no universal formula for sample size determination, and there will not be one. Sample size requirements arise from the combination of research question, study design, expected effect size, chosen significance level, desired statistical power, the unit of analysis, and the analytical approach. Each of these inputs is design-specific. The remedy is not to find a universal formula; it is to match the formula to the design.

This methodology guide consolidates the sample size guidance for the study designs that HPE researchers most commonly use. For each design, we present the underlying logic, formula, or rule of thumb, a worked example in an HPE context, and reporting expectations. The guide draws on foundational statistical references ^7^ ^–^ ^11^ and on HPE-specific methodological work. ^1^ ^,^ ^12^ ^,^ ^13^

## Objectives

This methodology guide consolidates the sample-size logic currently dispersed across statistical textbooks and discipline-specific methodological papers into a single, design-stratified reference for HPE researchers. Its organising principle is that sample size in HPE research is a design-specific judgment rather than a formula-specific calculation: the required sample follows from the research question, study design, expected effect size, significance level, power, unit of analysis, and analytical approach, and the appropriate response is to match the calculation to the design rather than to search for a universal formula.

## Main body

### Foundational principles

Every sample-size calculation, regardless of design, rests on a small set of decisions that must be made before any formula is invoked. Three of these are statistical parameters supplied to the calculation: effect size the study aims to detect, the significance level (α), and the statistical power (1-β). ^14^ Two further decisions are structural rather than parametric: the analytical model that will be applied to the data and the unit of analysis at which that model operates. The remainder of this section addresses each of these in turn; subsequent sections show how their interaction is design-specific.

The effect size is the magnitude of the difference, association, or change that the study aims to detect. It is the most consequential and poorly handled component of the sample size calculation in HPE. ^14^ Effect sizes are typically expressed in different metrics depending on outcome type: standardised mean differences (Cohen’s d) for continuous outcomes, correlations (Pearson’s r) for associations, and odds ratios or risk ratios for binary outcomes. Cohen’s original conventions classify d values of 0.2, 0.5, and 0.8 as small, medium, and large, ^7^ but these labels are field-dependent. In HPE, the empirical effect-size distribution depends critically on the comparator. Comparisons of educational interventions against no intervention yield large pooled effects (typically d ≈ 0.7–1.0). ^2^ Comparisons against traditional or non-simulation instruction yield small-to-moderate effects (typically d ≈ 0.4–0.7). ^15^ Comparisons of two active interventions against each other yield distinctly smaller effects, often d ≈ 0.1–0.3, and it is in this class of studies that the most severe under-powering is documented. ^1^ Investigators planning HPE studies should anchor their expected effect size to the specific comparator class of the proposed design, not to a generic small/medium/large convention. ^16^

Significance level (alpha) is the probability of falsely rejecting a true null hypothesis, conventionally set at 0.05. Statistical power (1-β) is the probability of correctly rejecting a false null hypothesis, conventionally set at 0.80. ^7^ ^,^ ^10^ Cohen’s rationale for the 0.80 default was explicit: it reflects an implicit cost ratio in which Type I errors are treated as approximately four times more serious than Type II errors (β = 4α = 0.20), and this 4-to-1 weighting was offered as a reasonable default for behavioural research, not as a universal rule. Departures from the 0.05/0.80 defaults are appropriate in specific circumstances. Where multiple comparisons are planned, a more stringent α (typically Bonferroni- or Holm-adjusted) is required to preserve the family-wise error rate. Where the consequences of missing a true effect are severe, for example, pivotal trials of educational interventions intended to inform policy or curriculum change investigators may target a power of 0.90 or 0.95. ^10^ The chosen α and power should be stated and justified, not left implicit.

The structure of the analysis determines which formula applies. A two-group comparison of means at a single time point uses the t-test sample size formula; the same data collected pre- and post-intervention within the same students uses a paired-samples or repeated-measures formula; the same data collected across clusters (classrooms, programs, hospitals) uses a cluster-randomized trial formula. The unit of analysis must match the unit of randomization or sampling. Treating clustered data as if observations were independent, or treating repeated measures as if they were independent observations, is a category error that invalidates both the sample size calculation and the inference built on it.

The general principle that follows is that sample size is downstream of design, not upstream of it. Investigators who choose a sample size before specifying the design, the analysis, and the expected effect size are guessing. Investigators who specify these first and derive the sample size from them are doing methodology. Every subsequent section in this guide operationalizes this principle for a specific design.

### Steps in sample size determination

Although the appropriate formula is design-specific, the sequence of decisions that precedes it is common to every study. The following steps operationalise the foundational principles above into a reproducible workflow. ^13^ Working through them in order prevents the most frequent failure mode in HPE research, in which a sample size is selected first and a justification is reconstructed afterwards. ^1^ ^,^ ^5^ The design-specific formulas, rules of thumb, and worked examples follow in Table 1 and the sections thereafter.

**Table 1.** Roadmap demonstrating design-specific inputs, formulas or rules, and example required sample sizes.

Study design	Key inputs	Formula or rule	Example required N
Two-group, means	α, power, σ, Δ (or d)	n per group = (Z₁₋α/₂ + Z₁₋β) ^2^ × 2/d ^2^	63 per group for d = 0.5, α = 0.05, power = 0.80
Two-group, proportions	α, power, p₁, p₂	n per group = (Z₁₋α/₂ + Z₁₋β) ^2^ × [p₁(1 − p₁) + p₂(1 − p₂)] / (p₁ − p₂) ^2^	167 per group for p₁ = 0.50, p₂ = 0.65, α = 0.05, power = 0.80
Paired (pre-post)	α, power, d_z (standardized paired effect)	n = (Z₁₋α/₂ + Z₁₋β) ^2^/d_z ^2^ + df correction	34 for d_z = 0.5, α = 0.05, power = 0.80
Cluster-randomized	Individual n inflated by design effect; ICC; cluster size m	DE = 1 + (m − 1) × ICC; required n = individual n × DE	DE = 1.95 for m = 20 and ICC = 0.05 (almost doubles required N)
Survey (proportion estimation)	Confidence level, expected p, margin of error E	n = Z ^2^ × p(1 − p) /E ^2^; finite population correction if applicable	384 for p = 0.5, E = 0.05, 95% CI; 218 with N = 500 correction.
Pilot or feasibility	Parameter being estimated, desired precision	Rule of thumb; reliability-specific rules	12 per group for variance/effect-size pilots; 30 for Cronbach α
Qualitative	Aim, sample specificity, theory use, dialogue quality, analysis strategy	Information power; empirical saturation	9 to 20 interviews typical for homogeneous groups
Simulation-based education	α, power, realistic d (typically 0.2 to 0.4 in this field)	Two-group or paired formula with field-appropriate d	About 175 per group for d = 0.3; about 393 per group for d = 0.2 (both at α = 0.05, power = 0.80)
Instrument validation	Psychometric property, expected reliability, model complexity	Cronbach α; COSMIN guidance for measurement properties	~30 for Cronbach α; ≥ 200 for confirmatory factor analysis/SEM

Z₁₋α/₂ and Z₁₋β are the standard normal critical values for the chosen alpha and power. σ is the standard deviation of the outcome, Δ is the mean difference, d is Cohen’s standardized mean difference, d_z is Cohen’s d for paired samples, ICC is the intracluster correlation coefficient, m is the average cluster size, p is the expected proportion, and E is the acceptable margin of error. Sample size estimates with fractional values were rounded up to the nearest whole number, as sample size must be expressed in whole participants.

### Step 1: Define the research question and the primary outcome

State the single primary outcome on which the study will be powered, together with its measurement scale (continuous, binary, ordinal, or time-to-event). Secondary outcomes do not drive the calculation and should not be used to power the study. ^17^

### Step 2: Identify the study design and the unit of analysis

Fix the design (two-group, pre–post, cluster-randomized, survey, Delphi, qualitative, or validation) and the unit at which observations are independent. The unit of analysis must match the unit of randomization or sampling; a mismatch invalidates both the calculation and the inference built on it. ^4^

### Step 3: Select the analytical model

Choose the test or model that matches the design and the outcome type (independent or paired t-test, test of two proportions, mixed-effects model, factor analysis, and so on). The formula follows from the model, not the reverse.

### Step 4: Determine the expected effect size, and anchor it to a credible source

This is the most consequential and most poorly handled input in HPE sample size calculation. ^14^ The expected effect size should be drawn from one of two sources: (i) a pilot or feasibility study conducted in a comparable population, or (ii) a prior published study, or a meta-analysis, of a comparable intervention, comparator class, population, and outcome. When a parent article is used, the effect size and its variability should be extracted from a study whose comparator matches the planned comparator, because effect sizes in HPE depend strongly on the comparator: interventions against no intervention yield large effects, those against active or traditional instruction yield small-to-moderate effects, and active-versus-active comparisons yield the smallest effects. ^1^ ^,^ ^2^ ^,^ ^15^ Where several estimates are available, a pooled or conservative (lower-bound) value is preferable to the largest reported effect. Pilot studies are well suited to estimating variability and feasibility but are too small to provide stable effect-size point estimates; their point estimates should not be used directly to power a definitive trial. ^18^ ^,^ ^19^ The default Cohen conventions (small, medium, large) are a last resort, not a substitute for a comparator-matched estimate. ^7^ ^,^ ^16^

### Step 5: Set the significance level and power, and justify any departure

Use α = 0.05 and power = 0.80 unless there is a reason to do otherwise; adjust α for multiplicity, and raise power to 0.90–0.95 for pivotal studies intended to inform policy or curriculum change. ^7^ ^,^ ^10^ State both values explicitly rather than leaving them implicit.

### Step 6: Account for design features

Inflate for clustering using the design effect and the intracluster correlation ^20^; use the within-subject correlation for paired and repeated-measures designs ^21^; adjust for unequal allocation; and correct for multiple comparisons where these are planned.

### Step 7: Apply the design-appropriate formula or rule to obtain the sample size at analysis

Use the formula or rule of thumb for the chosen design ( Table 1), or specialised software (for example, G*Power, PASS, or R) for designs without a closed-form solution. ^22^

### Step 8: Inflate for expected attrition

Convert the analysis sample size into a recruitment target using the expected drop-out rate, applying the inflation factor described in the section on attrition below. ^23^

### Step 9: Report the calculation transparently

Record the primary outcome, the design, the analytical model, the assumed effect size and its source, α, power, any design-effect or correlation adjustments, the resulting sample size, and the attrition inflation, in line with the SAMPL guidelines. ^17^

### Sample size requirements by study design: A quick reference

Table 1 summarizes the design-specific inputs, formulas or rules, and example required sample sizes detailed in the subsequent sections. The table is a roadmap. The accompanying text provides the underlying logic, common errors, and reporting expectations for each design.

### Two-group comparison of means and proportions

The two-group comparison is the workhorse design in educational intervention research, and standard sample size formulas apply most directly in this case. For the comparison of means in two independent groups (for example, examination scores between a new and a traditional curriculum), the required sample size (n) per group is approximately:

$$n={\left(Z\left(1-\alpha /2\right)+Z\left(1-\beta \right)\right)}^{2}\times {\text{2σ}}^{2}/\Delta^{2}$$

where σ is the expected standard deviation of the outcome, and Δ is the expected mean difference. ^8^ The formula is the Z-approximation form; for small samples, exact calculations based on the noncentral t distribution give marginally larger values and should be used when n per group is below approximately 30. Reformulating using Cohen’s d (the standardised mean difference, d = Δ/σ), the same formula simplifies to:

$$n={\left(Z\left(1-\alpha /2\right)+Z\left(1-\beta \right)\right)}^{2}\times 2/d^{2}$$

For α = 0.05, power = 0.80, and a medium effect size of d = 0.5, this yields approximately 63 participants per group. For a small effect size (d = 0.2), the required sample size sharply increased to approximately 393 per group. The relationship between effect size and required sample is inverse-quadratic: halving the expected effect quadruples the required sample. Investigators planning studies with unequal allocation (for example, comparing a single intervention cohort against multiple historical control cohorts) should note that the formula above assumes a 1:1 allocation; for a ratio r = n₂/n₁, the intervention-group size becomes n₁ = (Z₁₋α/₂ + Z₁₋β) ^2^ × σ ^2^(1 + 1/r) /Δ, ^2^ and the total sample is minimised at r = 1.

For comparison of proportions in two groups (for example, pass rates between two assessment formats), the required sample size per group is approximately:

$$n={\left(Z\left(1-\alpha /2\right)+Z\left(1-\beta \right)\right)}^{2}\times \left[p₁\left(1-p₁\right)+p₂\left(1-p₂\right)\right]/{\left(p₁-p₂\right)}^{2}$$

where p₁ and p₂ are the expected proportions in the two groups. ^9^ To detect a difference between p₁ = 0.50 and p₂ = 0.65 at alpha = 0.05 and power = 0.80, approximately 167 participants per group are required.

The most common error in this design is to assume a larger effect size than the literature supports. An assumed d = 0.5 with an actual d = 0.3 yields a study with approximately 40% power to detect the true effect, meaning the study is more likely to miss the true effect than to find it. A pilot study or a systematic review of effect sizes from comparable interventions should inform the assumed effect size, not Cohen’s default conventions.

### Pre-post and repeated-measures designs

When the same participants are measured before and after an intervention, the relevant analysis is paired-samples (paired t-test, or analogously, repeated-measures ANOVA for multiple time points). The within-subject correlation between baseline and follow-up reduces the variance of the change score, which in turn reduces the required sample size compared with a between-group design at the same effect size. ^21^ The paired-samples sample size formula is:

$$n={\left(Z\left(1-\alpha /2\right)+Z\left(1-\beta \right)\right)}^{2}\times \sigma^{2}\_d/\Delta^{2}$$

where σ ^2^_d is the variance of the difference scores, and Δ is the expected mean change. The variance of difference scores is related to the variance of the original measurements and the within-subject correlation r by σ ^2^_d = 2σ ^2^(1 − r). When r is high (for example, r = 0.7), σ ^2^_d is much smaller than 2σ ^2^, and the required sample size is correspondingly smaller. Using the standardized paired effect size d_z = Δ/σ_d (Cohen’s d for paired samples, as implemented in G*Power and equivalent software), ^22^ a study with d_z = 0.5, alpha = 0.05, and power = 0.80 requires approximately 34 participants.

Pre-post designs are common in educational interventions because participants serve as their own controls. The corresponding sample size advantage requires that an analysis appropriate to paired data is used. Treating pre-post data with an independent-samples t-test, or analyzing only the post-intervention score and ignoring baseline, gives away the design’s statistical power. Repeated-measures designs with three or more time points use mixed-effects or ANOVA frameworks; sample size calculation in these designs depends on the assumed correlation structure across time points and is typically conducted using specialized software such as G*Power, PASS, or R packages.

### Cluster-randomized educational trials

Educational interventions are often delivered at the level of a classroom, a program, a hospital department, or a cohort, rather than to individual learners. When randomization occurs at the cluster level, sample size calculation must account for the dependence between observations within the same cluster. Treating clustered data as if observations were independent inflates the apparent precision and produces incorrect inference. ^4^

The sample size required for a cluster-randomized trial is the sample size for an individually-randomized trial inflated by the design effect (DE):

DE=1+(m−1)×ICC

where m is the average cluster size, and ICC is the intracluster correlation coefficient, which quantifies the degree to which observations within the same cluster are correlated. ^20^ For HPE interventions delivered at the level of a clinical site or a teaching program, ICC values typically range from 0.01 to 0.10. With an ICC of 0.05 and cluster size m = 20, the design effect is 1 + 19 × 0.05 = 1.95, almost doubling the required sample size compared with an individually-randomized design.

Cluster-randomized trials require careful specification of the number of clusters to randomize, which is typically the more important quantity than the number of individuals within clusters, because increasing cluster size yields diminishing returns once the design effect is fixed; the cluster size, which is often determined by the natural unit (a typical class, a typical residency cohort); the expected ICC for the outcome measured, which should be informed by pilot data or comparable studies, not assumed at zero; and the unit and timing of measurement, which must match the unit of randomization and the time scale of the intervention.

A common error in HPE cluster-randomized trials is to power the study based on the total number of individuals enrolled, ignoring the cluster structure. Studies that randomize four classrooms of 30 students each (total n = 120) and then analyze outcomes as if from 120 independent students are not statistically valid. The effective sample size, accounting for the ICC, may be closer to 40–60. Designs beyond the simple two-arm parallel cluster trial, including three-level designs (learners within clusters within institutions), stepped-wedge designs (in which clusters cross over from control to intervention at staggered time points), and longitudinal cluster designs, are covered in depth. ^11^

### Survey and questionnaire studies

The sample size for descriptive survey research depends on the target population, desired precision of estimates (margin of error), assumed prevalence of the characteristic of interest, and chosen confidence level. The standard formula for estimating the population proportion is as follows:

$$n=Z^{2}\times p\left(1-p\right)/E^{2}$$

where Z is the critical value for the chosen confidence level (1.96 for 95% confidence), p is the expected proportion, and E is the acceptable margin of error. ^24^ For p = 0.5 (the most conservative assumption) and E = 0.05 at 95% confidence, the required sample size was approximately 384 participants.

When the target population is finite, the finite population correction is applied as follows:

n_adjusted=n/(1+(n−1)/N)

where N is the total population. A survey of an entire residency program with N = 500 trainees requires approximately 218 respondents to estimate a proportion with 5% margin of error and 95% confidence, rather than 384.

Two common errors in HPE survey research are worth flagging. First, calculating sample size based on a planned response rate rather than on the population size and desired precision produces nonsensical targets. Response rate is a practical consideration in recruitment, not a parameter in sample size calculation. Second, surveys with multiple subgroup analyses require larger total samples to ensure adequate precision in each subgroup, because the margin of error widens as subgroups become smaller. A survey planned to estimate overall prevalence at 5% precision will have much wider precision when stratified by year of training, specialty, or institutional setting. ^13^

### Delphi and consensus studies

The Delphi technique and related consensus methods are widely used in HPE to develop competency frameworks, curricula, assessment blueprints, and quality indicators. Like qualitative research, a Delphi study is not driven by statistical power; the number of participants is an expert panel size rather than a sample drawn to detect an effect. The appropriate panel size depends primarily on the heterogeneity of the expertise required. A systematic review of Delphi studies used to select healthcare quality indicators reported a median panel of 17 members (interquartile range 11 to 31) and noted that more than half of the panels were multi-stakeholder. ^25^

As a practical guide, single-stakeholder or homogeneous panels (for example, a single specialty or discipline) typically function with approximately 10 to 30 experts, whereas multi-stakeholder or heterogeneous panels (for example, clinicians, educators, students, and patients) should be larger, often 30 or more and sometimes up to about 60 to 90, so that each stakeholder group is adequately represented. Panel size should be justified by the diversity of perspectives required and by the need to retain sufficient members across rounds, rather than by a fixed number. A Delphi sample size justification should state the panel composition, the target panel size and its rationale, the number of rounds, the criteria used to define consensus, and the approach to attrition between rounds. ^26^ Table 2 summarises these and other anchors for designs in which a power-based calculation does not apply.

**Table 2.** Minimum and typical recommended sample sizes for designs and methods in which a power-based calculation does not apply.

Method or design	Basis for the number	Minimum or typical sample
Pilot or feasibility study (estimating variance or effect size for a future trial)	Precision of the variance estimate; rule of thumb	≥12 per group
Pilot for questionnaire reliability (Cronbach’s α or test–retest)	Precision of the reliability coefficient	30–50
Delphi (single-stakeholder or homogeneous panel)	Representativeness and stability of consensus within one discipline	~10–30 experts (median ~ 17)
Delphi (multi-stakeholder or heterogeneous panel)	Adequate representation of each stakeholder group across rounds	≥30, commonly up to ~60–90
Qualitative interviews (homogeneous group)	Saturation or information power	9–20 interviews
Cronbach’s α estimation	Precision of the α coefficient	~30
Exploratory factor analysis	Subjects per item; factor loadings and communalities	≥10 per item (loading-dependent)
Confirmatory factor analysis or structural equation modelling	Model complexity; indicators per latent variable	≥200

Values are practical anchors drawn from methodological rules of thumb and from systematic reviews of panel and saturation sizes; they are starting points to be justified against the study’s stated purpose, not fixed thresholds.

### Pilot and feasibility studies

Pilot and feasibility studies are not powered to detect intervention effects. Their purpose is to estimate parameters needed for a subsequent fully-powered trial: recruitment rates, retention, intervention fidelity, outcome variability, intracluster correlation, or instrument performance. ^18^ Sample size calculation for pilots follows a different logic than sample size calculation for hypothesis-testing studies.

A widely cited rule of thumb of 12 participants per group was proposed for pilot studies, which was aimed at estimating effect size and variance for subsequent trials. ^19^ The rule is based on the precision with which the variance of the outcome can be estimated, not on the power to detect an effect. The rule applies when the purpose is parameter estimation for a future definitive trial.

For pilots aimed at testing questionnaire reliability, sample sizes between 30 and 50 were recommended, depending on the number of items and the desired precision of the reliability coefficient (Cronbach’s alpha or test-retest correlation). For questionnaires with 10 to 20 items targeting a Cronbach’s alpha of 0.80, approximately 30 participants typically provide stable estimates. ^27^

The defining feature of a properly reported pilot study is that the sample size justification is tied to the pilot’s stated purpose, not to a generic rule of thumb. A pilot designed to estimate recruitment rates may require different numbers than a pilot designed to estimate outcome variance, which in turn differs from a pilot designed to test instrument reliability. A pilot study sample size justification should specify the parameter being estimated, the desired precision, and the source of the rule or formula used.

A common error is to use pilot effect size estimates as the basis for the main trial sample size calculation. Pilot studies are too small to provide stable effect size estimates, and using their point estimates as the basis for main trial sample size calculation underestimates the required sample. ^18^

### Qualitative research

Qualitative research does not use sample size calculation in the statistical sense, because its purpose is not to estimate population parameters or test hypotheses against a null distribution. The relevant concept is saturation: the point at which additional data collection no longer yields new themes, codes, or insights. ^28^

Saturation as a sample size principle has been criticized for being vague and inconsistently applied. The concept of information power was proposed, which holds that the sample size required for a qualitative study depends on the study’s aim (broad or narrow), specificity of the sample (specific or sparse), use of established theory (applied or not), quality of dialogue (strong or weak), and analysis strategy (cross-case or case). ^29^ A study with a narrow aim, a specific sample, an established theoretical framework, strong interview dialogue, and case analysis can achieve information power with fewer participants than a study with a broad aim and a sparse sample.

Empirical work on saturation provides practical anchors. For studies aimed at characterizing the perspective of a relatively homogeneous group, the majority of themes typically appear within 12 interviews, and saturation is approached around 12 to 20 interviews. ^28^ A more recent systematic review of empirical saturation studies reported similar findings across diverse qualitative research traditions, with most studies reaching saturation between 9 and 17 interviews. ^30^

For HPE qualitative research, a defensible sample size justification combines an information-power assessment with reference to empirical saturation evidence relevant to the study design. Focus group studies have different saturation dynamics than in-depth interviews. A study comparing perspectives across distinct subgroups requires sufficient depth within each subgroup. A study aimed at theory development may require more interviews than a study aimed at descriptive characterisation.

The sample size justification should state the principle invoked (saturation, information power, theoretical sampling), the planned monitoring approach during data collection, and the stopping rule.

### Simulation-based education research

Simulation-based education (SBE) is a substantial body of HPE research with its own design features that affect sample size requirements. The systematic review of sample size adequacy in HPE research cited at the start of this guide ^1^ found that the majority of SBE studies were powered to detect only large effects, with median sample sizes in the range of 25 to 30 participants per arm. The systematic review documented that small effect sizes are common in SBE comparison studies, particularly when comparing two active interventions against each other.

The implication for SBE sample size is that the field’s typical effect sizes are small relative to the convention. Studies powered on a medium-effect assumption are routinely underpowered for the effects that actually exist in SBE research. A sample size calculation grounded in published SBE effect size literature (typically d = 0.2 to 0.4 for active-versus-active comparisons) yields substantially larger sample size targets than calculations grounded in Cohen’s medium-effect convention.

SBE studies also frequently involve multiple outcomes (cognitive, procedural, and attitudinal) and repeated measurements. Multiple-outcome studies require correction for multiple comparisons or a primary outcome specification, and repeated measurements should be analysed using methods appropriate for repeated measures. Sample size calculations should refer to the primary outcome on which the study is powered, rather than multiple secondary outcomes.

A defensible SBE sample size justification cites published effect size estimates from comparable interventions, identifies the primary outcome, specifies the analytical method (paired versus independent, single time point versus repeated), and reports the assumed alpha, power, and effect size.

### Instrument validation studies

The sample size for instrument validation studies depends on the psychometric property being estimated. Approximately 30 participants are required to achieve reasonable precision for an expected alpha of approximately 0.80 when estimating Cronbach’s alpha, with more participants required when the expected alpha is lower or the precision requirements are higher. ^31^ Similar sample sizes (30–50 participants) are typically required for test-retest reliability estimation.

For exploratory factor analysis, the rule of 10 participants per item is widely cited; however, more contemporary work suggests that the appropriate sample size depends on factor loadings, communalities, and the number of factors expected. The COnsensus-based Standards for the selection of health Measurement INstruments (COSMIN) guidelines provide structured guidance on sample size for measurement property studies and are the appropriate reference for HPE instrument validation work. ^32^

Confirmatory factor analysis and structural equation modelling typically require larger samples (≥ 200) and depend on model complexity, expected effect sizes for parameter estimation, and the number of indicators per latent variable. Investigators planning instrument validation should consult specialised psychometric resources rather than general sample size formulas. Sample size justifications for validation studies should reference COSMIN or comparable measurement-science guidance.

### Reporting sample size justification

A methods section should report sample size justification in sufficient detail that an independent reader can reproduce the calculation. The Statistical Analyses and Methods in the Published Literature (SAMPL) guidelines recommend that sample size reporting include the primary outcome on which the study is powered, the analytical method, the assumed effect size and its source, the chosen alpha and power, any adjustments for design effect or correlation, and the calculated required sample size. ^17^ For pilot and qualitative studies, the analogous information includes the parameter being estimated or the saturation or information-power principle applied the source for any rule of thumb invoked, and the planned monitoring approach during data collection.

Sample size justifications should also report what was actually achieved. Studies that fail to reach the planned sample size should report the final sample, the reason for the shortfall, and the implications for power. Studies that exceed the planned sample size should report the final sample and the rationale without retrospectively re-powering the analysis.

Common reporting deficiencies that this guide aims to help authors avoid include: stating a sample size without justification; citing a software output without parameters; citing a rule of thumb without naming the rule or its source; failing to specify the unit of analysis; calculating power on a secondary outcome when the analysis is reported on a primary outcome; and reporting power calculated retrospectively from observed data.

Reviewers and editors evaluating sample size justifications should check that the design, the analytical method, the assumed effect size, the chosen alpha and power, the unit of analysis, and the final sample size are all internally consistent. Inconsistency among these components is a common signal of sample size determination that was not done before data collection but was reconstructed afterward.

### Adjusting for expected attrition

Most HPE studies experience some loss of participants between recruitment and analysis. The sample size calculated from any of the formulas in this guide represents the number of participants required at analysis, not at recruitment. To account for expected attrition, the calculated sample size N should be inflated to a recruitment target N_d:

N_d=N/(1−d)

where d is the expected proportion lost to drop-out or non-compliance. For a study requiring N = 100 participants at analysis with an expected drop-out rate of 20% (d = 0.20), the recruitment target becomes N_d = 100/ (1–0.20) = 125. The choice of d should be informed by the attrition rates observed in comparable studies, the length and intensity of the intervention, and population characteristics. Sample size justification should report both the calculated N and inflated recruitment target N_d, along with the source of the assumed attrition rate. ^23^

### Use cases

This guide brings established sample size procedures together into one workflow organized by study design rather than proposing a new estimator, so formal validation through simulation or empirical accuracy testing does not apply. The formulas and rules it uses are already validated in the statistical literature cited throughout, including Cohen’s effect size conventions, the cluster design effect, the finite population correction, the information power approach, and COSMIN guidance. Each design section shows them applied through a worked example set in HPE practice, and the logic of the guide extends the authors’ earlier work on sample size in educational research.

The workflow is meant for use at the design stage: investigators identify the design and unit of analysis, choose the matching model, anchor the effect size to a source matched to the comparator, justify alpha and power, adjust for clustering or repeated measures, apply the formula, inflate for attrition, and report against SAMPL. Reviewers and editors can follow the same sequence as an appraisal checklist. Its value is to reduce the two documented failure modes, silent underpowering and post hoc justification, by giving the field one reference matched to the design. Peer review will be most useful in testing the worked examples and the effect size ranges suggested for each comparator class.

## Conclusions

Sample size determination in health professions education research is design-specific rather than formula-specific. The required sample size for a study follows from the research question, study design, expected effect size, chosen significance level and power, unit of analysis, and analytical approach. Each of these inputs is design-specific, and changing any of them changes the required sample size. The widespread search for a universal formula reflects a methodological misunderstanding that this guide has aimed to dispel.

The practical implication for HPE investigators is that sample size belongs at the design stage of a study, integrated with the choice of research question, study design, and analytical strategy, and not added as a post-hoc justification. The practical implication for reviewers and editors is that sample size justifications should be evaluated against the design they support, and not against generic conventions imported from other disciplines.

## AI disclosure

The authors used Claude (Anthropic; Claude Opus 4.x) to assist with language editing. All AI-assisted output was critically reviewed, verified, and edited by the authors, who take full responsibility for the originality, accuracy, and integrity of the content.

## Data Availability

No data are associated with this article. Not applicable. This article is a methodological guide and does not report primary empirical research; no reporting-guideline checklist applies.
