# Sample size determination across study designs in health professions education research: a methodology guide

**DOI:** 10.12688/f1000research.185853.2

**Published:** 2026-09-03

**Authors:** Waqar M Naqvi, Anusha Divvi, Shivashankar Kengadaran, Aishwarya A Pashine, Maheshkumar Baladaniya, Aditya Patel, Praveen Kumar Kandakurthi, Ramprasad Muthukrishnan

**Affiliations:** 1 Department of Health Professions Education, Faculty of Interdisciplinary Sciences, Datta Meghe Institute of Higher Education and Research, Wardha, India; 2 Public Health Dentistry, Indira Gandhi Institute of Dental Sciences, Sri Balaji Vidyapeeth University, Puducherry, Puducherry, India; 3 Department of Technical Medicine (Medical Simulation), Faculty of Interdisciplinary Sciences, Datta Meghe Institute of Higher Education and Research, Wardha, India; 4 Department of Physical Therapy, Neighborhood Physical Therapy PC, New York, USA; 5 Department of Conservative Dentistry and Endodontics, Sharad Pawar Dental College and Hospital, Datta Meghe Institute of Higher Education and Research, Wardha, India; 6 College of Health Sciences, Gulf Medical University, Ajman, Ajman, United Arab Emirates

**Keywords:** Sample size, Sample size calculation, Statistical power, Effect size, Research design, Health professions education

## Abstract

**Background:**

Sample size determination is one of the least reported elements in the methods sections of health professions education (HPE) research. Underpowered studies miss real effects, whereas overpowered studies waste participants and resources. Guidance remains fragmented across statistical textbooks and discipline-specific papers, with no unified reference for HPE researchers.

**Methods:**

We developed this guide as a structured narrative synthesis. Sources were identified through targeted database searching, citation chaining, and standard statistical texts, and selected against pre-specified criteria; where recommendations conflicted, precedence was given to the primary methodological source and to empirical evidence generated within HPE. Rather than proposing a universal formula, which does not exist, we organised sample-size logic by study design. We set out a stepwise workflow and emphasise that the expected difference should be anchored to previous evidence and to educational importance, with pilot work used to estimate variability, recruitment and attrition rather than the effect size itself. For each design we give the statistical rationale, the applicable formula or rule, a worked example with fully stated assumptions, and reporting expectations.

**Results:**

The designs covered include two-group comparisons of means and proportions, pre–post and repeated-measures designs, cluster-randomized educational trials, survey and questionnaire studies, Delphi and consensus studies, pilot and feasibility studies, qualitative enquiry, simulation-based education, and instrument validation. The guide concludes with a reporting checklist for the methods section, serving investigators, peer reviewers, and journal editors. Precision-based planning, software implementation, and designs beyond closed-form calculation are addressed separately.

**Conclusions:**

Sample size in HPE research is a design-specific judgment, not a formula-specific calculation. The numerical benchmarks reproduced here are illustrative anchors rather than methodological minima. This guide consolidates that judgment into a practical reference reflecting the methodological diversity of the field.

## Introduction

Sample size is one of the most consequential design decisions in any health professions education (HPE) study, yet it is routinely under-reported and under-justified. A systematic review of sample size adequacy in HPE research reported a median sample size of 25 per study and found that fewer than 1% had ≥80% statistical power to detect a small standardized effect (SMD > 0.2), while only about one in five could detect a large effect (SMD > 0.8). ^1^ This suggests that, under realistic effect sizes for educational interventions, a substantial proportion of the existing HPE evidence base lacks adequate statistical power. Underpowered studies have a low probability of detecting true effects, but they also have a low positive predictive value: when an underpowered study does report a statistically significant result, that result is more likely to be a false positive and, if genuine, is likely to be exaggerated in magnitude. ^2^

Sample size errors in HPE research fall into recognizable patterns. Reviews of methodological quality in medical and health professions education have documented that sample-size justifications are frequently absent, are restricted to a single number with no stated effect size or assumptions, or rely on rules of thumb that do not match the analytical model being used. ^3^ ^,^ ^4^ A particularly common error in cluster-delivered interventions is to treat learners within classrooms, programs, or clinical sites as independent observations, ignoring the intracluster correlation and overstating the effective sample size. ^5^ A different but equally common error is post hoc justification, calculating or describing power after data have been collected, a practice that is uninformative for inference, and the argument provides no useful information beyond the p-value itself. ^6^ An earlier review by some of the present authors documented variability in sample size practices in published educational research. ^7^ The current guide provides the design-driven methodological grounding needed to address that variability.

There is no universal formula for sample size determination, and there will not be one. Sample size requirements arise from the combination of research question, study design, expected effect size, chosen significance level, desired statistical power, the unit of analysis, and the analytical approach. Each of these inputs is design-specific. The remedy is not to find a universal formula; it is to match the formula to the design.

This methodology guide consolidates the sample size guidance for the study designs that HPE researchers most commonly use. For each design, we present the underlying logic, formula, or rule of thumb, a worked example in an HPE context, and reporting expectations. The guide draws on foundational statistical references ^8^ ^–^ ^12^ and on HPE-specific methodological work. ^1^ ^,^ ^13^ ^,^ ^14^

## Objectives

The objective of this guide is to give HPE investigators, peer reviewers, and editors a single design-stratified reference that can be applied at the point at which a study is being planned. Three specific aims follow from that objective. First, to set out a reproducible nine-step workflow that fixes the design, the analytical model, and the unit of analysis before any formula is invoked. Second, to provide, for each design commonly encountered in HPE, the applicable formula or rule, a worked example with fully stated assumptions, and the errors most often seen in that design. Third, to specify what a defensible sample size justification must report, in a form that can be used prospectively by authors and as an appraisal aid by reviewers and editors.

### Methods: how this guide was developed

This guide is a structured narrative synthesis rather than a systematic review or a formal consensus exercise, and readers should interpret its recommendations accordingly. The development process had four components.

We identified and synthesised evidence from three main sources. The first was the standard statistical literature on sample size determination, comprising widely used reference texts ^8^ ^–^ ^10^ ^,^ ^12^ and primary methodological papers for individual designs. ^11^ ^,^ ^15^ ^–^ ^19^ The second was HPE-specific and health-professions-specific methodological literature, identified by targeted searching of MEDLINE/PubMed and Google Scholar using combinations of the terms *sample size*, *statistical power*, *effect size*, *health professions education*, *medical education*, and design-specific terms ( *cluster randomised*, *Delphi*, *saturation*, *pilot study*, *factor analysis*), supplemented by forward and backward citation chaining from key papers. ^1^ ^,^ ^3^ ^,^ ^13^ The third was existing reporting guidance relevant to sample size, principally the SAMPL guidelines ^20^ and the CONSORT cluster extension. ^5^

Sources were retained if they (i) stated a sample size formula, rule, or planning principle in a form that could be applied prospectively; (ii) reported empirical evidence on sample size adequacy, effect size distributions, or saturation in health professions or closely allied educational research; or (iii) constituted authoritative reporting guidance for the relevant design. We did not appraise the methodological quality of included sources formally, and no dual independent selection was performed.

Where recommendations differed, we applied a fixed order of precedence: the primary methodological source in which a rule originated was preferred over secondary citations of it; empirical evidence generated within HPE was preferred over conventions imported from other disciplines; and, where a defensible range existed rather than a single value, we report the range and the conservative end of it rather than the most permissive figure. Where genuine methodological disagreement persists as it does for pilot study sizing and for saturation in qualitative work, we say so in the relevant section rather than presenting a single number as settled.

All worked examples were computed by applying the closed-form expressions given in the text under the assumptions stated in the footnote to Table 1, and were cross-checked against G*Power 3.1 ^21^ and the R package **pwr**. ^22^ Because the synthesis was not systematic, relevant sources may have been missed, and the selection reflects the authors’ collective judgment as methodologists and HPE researchers. The guide has not been subjected to a Delphi or other formal consensus process, and it does not carry the authority of a consensus statement. Its claim is to be a defensible, transparently sourced, design-stratified reference, not a definitive one. The exact settings and syntax required to reproduce every example in Table 1 are provided as Extended data, File S1.

**Table 1.** Roadmap of design-specific inputs, analyses, formulas or rules, example required sample sizes, and common pitfalls.

Study design	Key inputs	Primary analysis	Formula or rule	Example required N	Key assumption and common pitfall
Two-group, means	α, power, σ, Δ (or d)	Independent-samples t-test	n per group = (Z₁₋α/₂ + Z₁₋β) ^2^ × 2/d ^2^	63 per group for d = 0.5, α = 0.05, power = 0.80	Assumes equal variances and 1:1 allocation; pitfall is assuming a larger *d* than the comparator class supports
Two-group, proportions	α, power, p₁, p₂	Chi-square or two-proportion z-test	n per group = (Z₁₋α/₂ + Z₁₋β) ^2^ × [p₁(1 − p₁) + p₂(1 − p₂)] / (p₁ − p₂) ^2^	167 per group for p₁ = 0.50, p₂ = 0.65, α = 0.05, power = 0.80	Normal approximation, no continuity correction; pitfall is powering on a relative rather than absolute difference
Paired (pre-post)	α, power, d_z (standardized paired effect)	Paired t-test	n = (Z₁₋α/₂ + Z₁₋β) ^2^/d_z ^2^ + df correction	34 for d_z = 0.5, α = 0.05, power = 0.80	Requires *d*_z_, not between-group *d*; pitfall is analysing paired data with an independent-samples test
Cluster-randomized	Individual n; ICC; cluster size m	Mixed-effects model or cluster-level summary	DE = 1 + (m − 1) × ICC; required n = individual n × DE	DE = 1.95 for m = 20 and ICC = 0.05 (almost doubles required N)	Assumes equal cluster sizes; pitfall is powering on total individuals and ignoring the cluster structure
Survey (proportion estimation)	Confidence level, expected p, margin of error E	Descriptive estimation with confidence interval	n = Z ^2^ × p(1 − p) /E ^2^; finite population correction if applicable	384 for p = 0.5, E = 0.05, 95% CI; 218 with N = 500 correction.	Assumes simple random sampling; pitfall is confusing completed responses with invitations issued
Analytical cross-sectional survey	α, power, exposure prevalence, expected difference or OR	Chi-square, t-test, or regression	Two-group means or proportions formula applied to the comparison of interest	As for the corresponding two-group row	Powered on the association, not on prevalence; pitfall is using a prevalence formula for a comparative aim
Pilot or feasibility	Parameter being estimated, desired precision	Descriptive; parameter estimation	Rule of thumb; reliability-specific rules	12 per group for variance/effect-size pilots; 30 for Cronbach α	Not powered for effect detection; pitfall is using the pilot effect estimate to power the definitive trial
Qualitative	Aim, sample specificity, theory use, dialogue quality, analysis strategy	Thematic or framework analysis	Information power; empirical saturation	9 to 20 interviews typical for homogeneous groups	Depends on homogeneity and interview depth; pitfall is citing saturation without a stopping rule
Delphi and consensus	Panel composition, stakeholder groups, rounds, consensus criteria	Descriptive; predefined consensus threshold	Panel size justified by diversity of expertise required	≈10–30 experts (homogeneous); ≥30, up to ≈60–90 (multi-stakeholder)	Not a power calculation; pitfall is reporting panel size without attrition across rounds
Simulation-based education	α, power, realistic d (typically 0.2 to 0.4 in this field)	Independent or paired t-test; repeated-measures model	Two-group or paired formula with field-appropriate d	≈175 per group for *d* = 0.3; ≈393 per group for *d* = 0.2	Field effect sizes are small; pitfall is powering on Cohen’s medium-effect convention
Instrument validation	Psychometric property, expected reliability, model complexity	Cronbach’s α; EFA; CFA/SEM	Reliability precision rules; COSMIN guidance for measurement properties	~30 for Cronbach α; ≥200 for confirmatory factor analysis/SEM	Loadings and communalities matter more than N:item ratios; pitfall is applying a fixed 10:1 rule regardless of model
Precision-based (any design)	Confidence level, σ or p, target interval half-width E	Estimation with confidence interval	n = (Z _1−α/2_ × σ/E) ^2^ for a mean; n per group = 2Z _1−α/2_^2^/E _d_^2^ for an SMD	97 for ±2 marks with σ = 10; 193 per group for an SMD to within ±0.20	Estimation, not testing; pitfall is reporting power for a study designed to estimate

## Main body

### Foundational principles

Every sample-size calculation, regardless of design, rests on a small set of decisions that must be made before any formula is invoked. Three of these are statistical parameters supplied to the calculation: effect size the study aims to detect, the significance level (α), and the statistical power (1-β). ^23^ Two further decisions are structural rather than parametric: the analytical model that will be applied to the data and the unit of analysis at which that model operates. The remainder of this section addresses each of these in turn; subsequent sections show how their interaction is design-specific.

***The expected effect size***

The effect size is the magnitude of the difference, association, or change that the study aims to detect. It is the most consequential and poorly handled component of the sample size calculation in HPE. ^23^ Effect sizes are typically expressed in different metrics depending on outcome type: standardised mean differences (Cohen’s d) for continuous outcomes, correlations (Pearson’s r) for associations, and odds ratios or risk ratios for binary outcomes.

Cohen’s original conventions classify d values of 0.2, 0.5, and 0.8 as small, medium, and large, ^8^ but these labels are field-dependent. In HPE, the empirical effect-size distribution depends critically on the comparator. Comparisons of educational interventions against no intervention yield large pooled effects (typically d ≈ 0.7–1.0). ^3^ Comparisons against traditional or non-simulation instruction yield small-to-moderate effects (typically d ≈ 0.4–0.7). ^24^ Comparisons of two active interventions against each other yield distinctly smaller effects, often d ≈ 0.1–0.3, and it is in this class of studies that the most severe under-powering is documented. ^1^ Investigators planning HPE studies should anchor their expected effect size to the specific comparator class of the proposed design, not to a generic small/medium/large convention. ^25^

***Educational importance, statistical significance, and the credibility of published effect sizes***

Three further considerations should temper the choice of effect size.

First, the effect size used to power a study should represent the smallest difference that would matter educationally, not the smallest difference that could be detected or the largest that could plausibly be claimed. There is no HPE equivalent of the minimal clinically important difference that applies across outcomes, but the general finding that a change of approximately half a standard deviation corresponds to a perceptible difference on many patient-reported measures ^26^ offers a useful starting anchor where no outcome-specific value exists. Investigators should state the minimally important educational difference they are targeting and the basis for it: expert judgment, prior literature, the properties of the assessment instrument, or the decision the result is intended to inform.

Second, statistical significance and educational significance are distinct. A statistically significant improvement of one percentage point on a summative examination in a large cohort may have no implication for curriculum design, while a non-significant difference of a full grade in a small cohort may be highly consequential and merely underpowered. Sample size planning should therefore begin from the magnitude of change that would alter practice, and results should be reported with effect estimates and confidence intervals rather than p-values alone.

Third, published effect sizes should be treated as upper bounds rather than unbiased estimates. Selective publication of statistically significant results, combined with the low power typical of the HPE literature, inflates the effect sizes that appear in print: in a low-powered literature, the effects that reach significance are systematically the overestimates. ^2^ Where a meta-analysis is available, a pooled estimate is preferable to any single study; where only individual studies are available, the lower bound of the reported confidence interval is a more defensible planning value than the point estimate.

***Significance level and power***

The significance level (α) is the probability of falsely rejecting a true null hypothesis, conventionally set at 0.05. Statistical power (1-β) is the probability of correctly rejecting a false null hypothesis, conventionally set at 0.80. ^8^ ^,^ ^11^ Cohen’s rationale for the 0.80 default was explicit: it reflects an implicit cost ratio in which Type I errors are treated as approximately four times more serious than Type II errors (β = 4α = 0.20), and this 4-to-1 weighting was offered as a reasonable default for behavioural research, not as a universal rule.

Departures from the 0.05/0.80 defaults are appropriate in specific circumstances. Where multiple comparisons are planned, a more stringent α (typically Bonferroni- or Holm-adjusted) is required to preserve the family-wise error rate. Where the consequences of missing a true effect are severe, for example, pivotal trials of educational interventions intended to inform policy or curriculum change investigators may target a power of 0.90 or 0.95. ^11^ The chosen α and power should be stated and justified, not left implicit and the direction of the test (one- or two-sided) should be stated with them; two-sided testing is the default and a one-sided test requires explicit justification.

***Planning for precision rather than power***

Power-based planning asks how many participants are needed to detect a specified effect. Precision-based planning asks a different and often more useful question: how many participants are needed to estimate the effect with an acceptable degree of certainty, expressed as the width of its confidence interval. ^11^ ^,^ ^27^ The two approaches are complementary, and precision-based planning is particularly appropriate in HPE for descriptive studies, for programme evaluations where the question is “how large is the effect” rather than “is there an effect”, and for studies whose results will be pooled in a future meta-analysis.

For estimating a single mean with a two-sided 95% confidence interval of half-width *E*,

$$n={\left(Z_{1-\alpha /2}\times \sigma /E\right)}^{2}$$

So, estimating a mean examination score to within ±2 marks, where the standard deviation is 10 marks, requires approximately 97 participants. For estimating a difference between two independent means expressed on the standardised scale, with a 95% confidence interval of half-width *E*_d_,

$$n\;\mathrm{per}\;\text{group}=2\times {Z_{1-\alpha /2}}^{2}/{E_{d}}^{2}$$

So, estimating a standardised mean difference to within ±0.20 requires approximately 193 participants per group, appreciably more than the 63 per group required merely to detect *d* = 0.5. The formula for estimating a proportion to within a specified margin of error is given in the section on survey studies and is the most familiar application of this logic in HPE.

Two implications follow. First, a study sized only to detect an effect will usually produce a confidence interval too wide to characterise its magnitude, which is why so many adequately powered educational studies nonetheless conclude that further research is required. Second, where a study is designed to estimate rather than to test, the sample size justification should report the target interval width and the assumed variability, not a power figure.

***The analytical model and the unit of analysis***

The structure of the analysis determines which formula applies. A two-group comparison of means at a single time point uses the t-test sample size formula; the same data collected pre- and post-intervention within the same students uses a paired-samples or repeated-measures formula; the same data collected across clusters (classrooms, programs, hospitals) uses a cluster-randomized trial formula.

The unit of analysis must match the unit of randomization or sampling. Treating clustered data as if observations were independent, or treating repeated measures as if they were independent observations, is a category error that invalidates both the sample size calculation and the inference built on it.

The general principle that follows is that sample size is downstream of design, not upstream of it. Investigators who choose a sample size before specifying the design, the analysis, and the expected effect size are guessing. Investigators who specify these first and derive the sample size from them are doing methodology. Every subsequent section in this guide operationalizes this principle for a specific design.

### Steps in sample size determination

**Figure 1.**
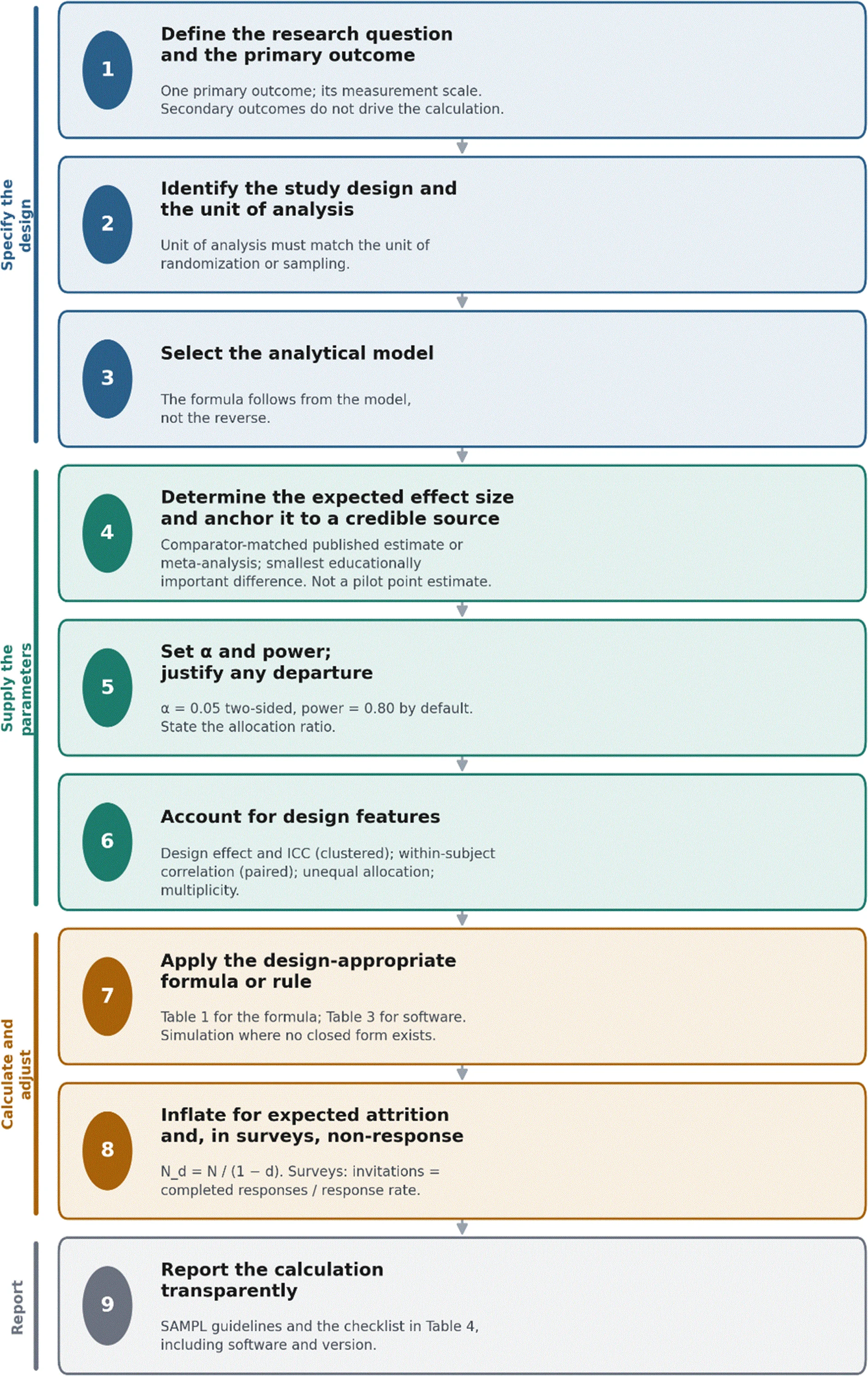
The nine-step sample size determination workflow.

Although the appropriate formula is design-specific, the sequence of decisions that precedes it is common to every study. The following steps operationalise the foundational principles above into a reproducible workflow ( Figure 1). ^14^ Working through them in order prevents the most frequent failure mode in HPE research, in which a sample size is selected first and a justification is reconstructed afterwards. ^1^ ^,^ ^6^ The design-specific formulas, rules of thumb, and worked examples follow in Table 1 and the sections thereafter.

### Step 1: Define the research question and the primary outcome

State the single primary outcome on which the study will be powered, together with its measurement scale (continuous, binary, ordinal, or time-to-event). Secondary outcomes do not drive the calculation and should not be used to power the study. ^20^

### Step 2: Identify the study design and the unit of analysis

Fix the design (two-group, pre–post, cluster-randomized, survey, Delphi, qualitative, or validation) and the unit at which observations are independent. The unit of analysis must match the unit of randomization or sampling; a mismatch invalidates both the calculation and the inference built on it. ^5^

### Step 3: Select the analytical model

Choose the test or model that matches the design and the outcome type (independent or paired t-test, test of two proportions, mixed-effects model, factor analysis, and so on). The formula follows from the model, not the reverse.

### Step 4: Determine the expected effect size, and anchor it to a credible source

As set out in the section on foundational principles above, the expected effect size should be drawn from a prior published study or meta-analysis whose comparator, population, and outcome match those planned, and should represent the smallest difference of educational importance. Where several estimates are available, a pooled or conservative (lower-bound) value is preferable to the largest reported effect. ^1^ ^,^ ^3^ ^,^ ^24^ Pilot studies are well suited to estimating variability, recruitment and attrition but are too small to provide stable effect-size point estimates; their point estimates should not be used directly to power a definitive trial. ^28^ ^,^ ^29^ The default Cohen conventions are a last resort, not a substitute for a comparator-matched estimate. ^8^ ^,^ ^25^

### Step 5: Set the significance level and power, and justify any departure

Use α = 0.05 (two-sided) and power = 0.80 unless there is a reason to do otherwise; adjust α for multiplicity, and raise power to 0.90–0.95 for pivotal studies intended to inform policy or curriculum change. ^8^ ^,^ ^11^ State both values, and the allocation ratio, explicitly rather than leaving them implicit.

### Step 6: Account for design features

Inflate for clustering using the design effect and the intracluster correlation ^15^; use the within-subject correlation for paired and repeated-measures designs ^16^; adjust for unequal allocation; and correct for multiple comparisons where these are planned.

### Step 7: Apply the design-appropriate formula or rule to obtain the sample size at analysis

Use the formula or rule of thumb for the chosen design ( Table 1), or specialised software ( Table 3) for designs without a closed-form solution. ^21^

### Step 8: Inflate for expected attrition and, in surveys, for non-response

Convert the analysis sample size into a recruitment target using the expected drop-out rate, applying the inflation factor described in the section on attrition below. ^30^

### Step 9: Report the calculation transparently

Record the primary outcome, the design, the analytical model, the assumed effect size and its source, α, power, allocation ratio, any design-effect or correlation adjustments, the resulting sample size, and the attrition inflation, and the software used in line with the SAMPL guidelines and the checklist in Table 4. ^20^

### Sample size requirements by study design: A quick reference

**Figure 2.**
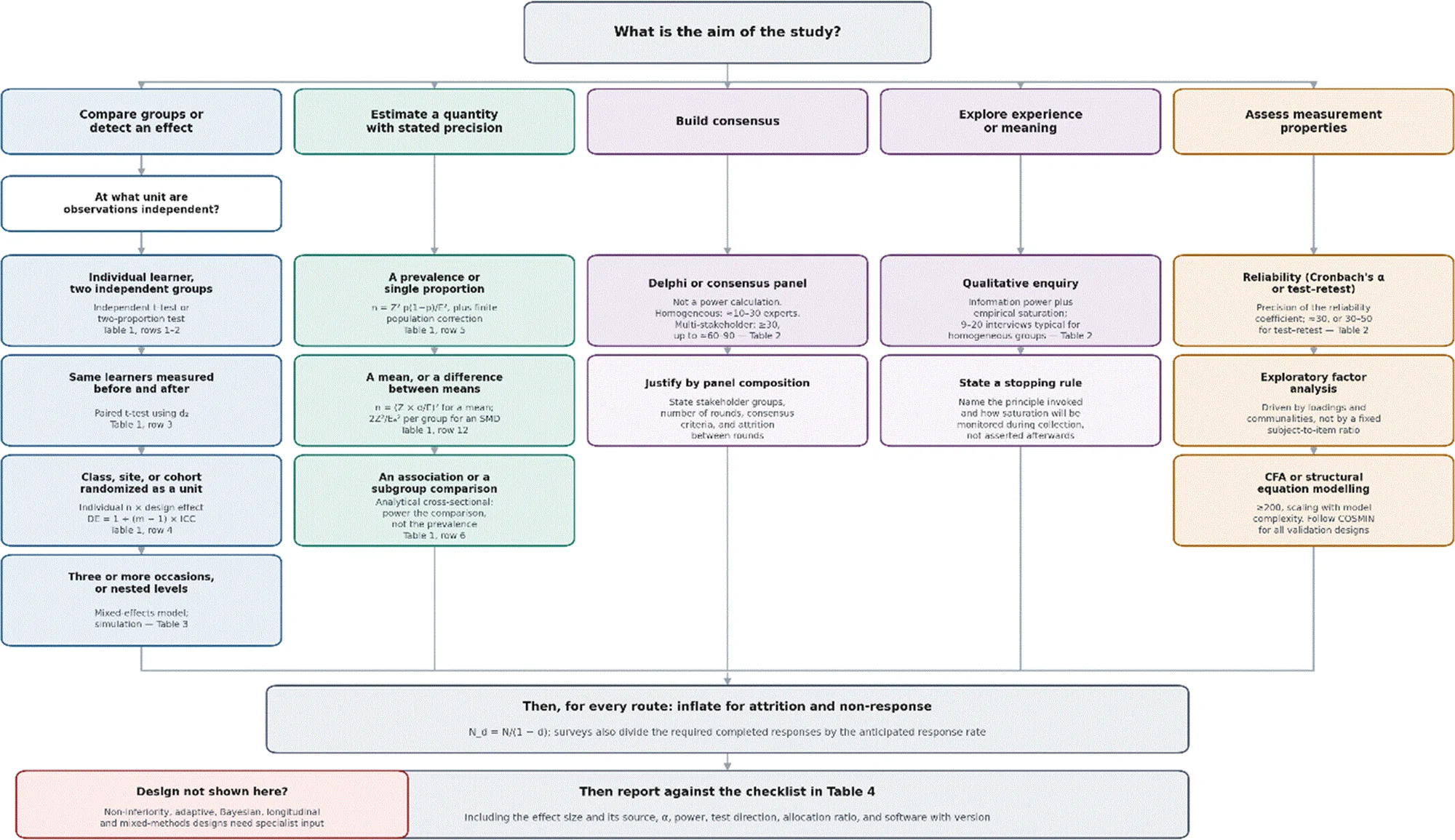
Decision tree for selecting a sample size method by study design and unit of analysis.

Table 1 summarizes the design-specific inputs, primary analyses, formulas or rules, example required sample sizes, and characteristic pitfalls detailed in the subsequent sections. Figure 2 presents the same material as a decision tree, mapping the design and unit of analysis onto the appropriate method. Both are roadmaps. The accompanying text provides the underlying logic, common errors, and reporting expectations for each design.

*Assumptions for all worked examples in* *Table 1* *, unless otherwise stated:* α = 0.05, two-sided; power = 0.80; 1:1 allocation; complete data with no attrition inflation applied (see the section on attrition); no adjustment for multiplicity; simple random sampling for survey rows; and, for the two-group means rows, equal variances in the two groups.

*Derivation and software.* The values above were computed from the closed-form expressions in the “Formula or rule” column, which use the normal approximation. They were then cross-checked against G*Power 3.1 ^21^ and the R package **pwr**, ^22^ which use exact solutions and therefore return marginally larger values in some cases. The differences are small but real, and are reported here rather than concealed, because a guide that asks investigators to report their software must show what difference the software makes: for two independent means, 63 per group by the normal approximation against 64 by the exact noncentral *t* at *d* = 0.5, 175 against 176 at *d* = 0.3, and 393 against 394 at *d* = 0.2; for two proportions, 167 per group by the closed-form expression against 170 using Cohen’s arcsine-transformed effect size *h* = 0.305 as implemented in pwr.2p.test (the closed-form value of 167 delivers 79.5% rather than 80.0% power on exact calculation); and for the paired example, 34 by both routes. The survey figure of 384 follows the conventional rounding of 384.16 used in the source table; ^31^ strict rounding up gives 385. Where a difference of this magnitude would matter to a study, the exact solution should be preferred and the software named. Full settings and syntax for every example are provided as Extended data, File S1.

A decision tree for selecting a sample size method by study design and unit of analysis is depicted in Figure 2. The tree routes an investigator from the aim of the study (estimation, comparison, consensus, exploration, or measurement) through the unit of analysis to the applicable formula, rule, or software route.

### Two-group comparison of means and proportions

The two-group comparison is the workhorse design in educational intervention research, and standard sample size formulas apply most directly in this case. For the comparison of means in two independent groups (for example, examination scores between a new and a traditional curriculum), the required sample size ( *n*) per group is approximately:

$$n={\left(Z_{1-\alpha /2}+Z_{1-\beta }\right)}^{2}\times {\text{2σ}}^{2}/\Delta^{2}$$

where σ is the expected standard deviation of the outcome and Δ is the expected mean difference. ^9^ This is the Z-approximation form; exact calculations based on the noncentral t distribution give marginally larger values and should be used when n per group is below approximately 30. Reformulating using Cohen’s *d* (the standardised mean difference, *d* = Δ/σ), the same formula simplifies to:

$$n={\left(Z_{1-\alpha /2}+Z_{1-\beta }\right)}^{2}\times 2/d^{2}$$

For α = 0.05 (two-sided), power = 0.80, and a medium effect size of *d* = 0.5, this yields approximately 63 participants per group by the Z-approximation, or 64 per group by the exact noncentral t solution implemented in G*Power. For a small effect size ( *d* = 0.2), the required sample size rises sharply to approximately 393 per group. The relationship between effect size and required sample is inverse-quadratic: halving the expected effect quadruples the required sample.

Investigators planning studies with unequal allocation (for example, comparing a single intervention cohort against multiple historical control cohorts) should note that the formula above assumes 1:1 allocation. For an allocation ratio *r* = *n*_2_/ *n*_1_, the intervention-group size becomes:

$$n_{1}={\left(Z_{1-\alpha /2}+Z_{1-\beta }\right)}^{2}\times \sigma^{2}\times \left(1+1/r\right)/\Delta^{2}$$

with *n*_2_ = r × *n*_1_, and the total sample size is minimised at *r* = 1.

For comparison of proportions in two groups (for example, pass rates between two assessment formats), the required sample size per group is approximately:

$$n={\left(Z_{1-\alpha /2}+Z_{1-\beta }\right)}^{2}\times \left[p_{1}\left(1-p_{1}\right)+p_{2}\left(1-p_{2}\right)\right]/{\left(p_{1}-p_{2}\right)}^{2}$$

where p _1_ and p _2_ are the expected proportions in the two groups. ^10^ To detect a difference between p _1_ = 0.50 and p _2_ = 0.65 at α = 0.05 and power = 0.80, approximately 167 participants per group are required. Applying a continuity correction increases this figure and is the more conservative choice for small expected samples.

The most common error in this design is to assume a larger effect size than the literature supports. An assumed *d* = 0.5 with an actual *d* = 0.3 yields a study with approximately 40% power to detect the true effect, meaning the study is more likely to miss the true effect than to find it. A systematic review of effect sizes from comparable interventions, or a comparator-matched published estimate, should inform the assumed effect size, not Cohen’s default conventions.

### Pre-post and repeated-measures designs

When the same participants are measured before and after an intervention, the relevant analysis is paired-samples (paired t-test, or analogously, repeated-measures ANOVA for multiple time points). The within-subject correlation between baseline and follow-up reduces the variance of the change score, which in turn reduces the required sample size compared with a between-group design at the same effect size. ^21^ The paired-samples sample size formula is:

$$n={\left(Z_{1-\alpha /2}+Z_{1-\beta }\right)}^{2}\times \sigma_{d}^{2}/\Delta^{2}$$

where σ ^2^_d_ is the variance of the difference scores and Δ is the expected mean change. The variance of difference scores is related to the variance of the original measurements and the within-subject correlation r by σ ^2^_d_ = 2σ ^2^(1 − r). When r is high (for example, r = 0.7), σ ^2^_d_ is much smaller than 2σ ^2^, and the required sample size is correspondingly smaller. Using the standardized paired effect size *d*_z_ = Δ/σ _d_ (Cohen’s *d* for paired samples, as implemented in G*Power and equivalent software), ^21^ a study with *d*_z_ = 0.5, α = 0.05, and power = 0.80 requires approximately 34 participants.

Pre-post designs are common in educational interventions because participants serve as their own controls. The corresponding sample size advantage requires that an analysis appropriate to paired data is used. Treating pre-post data with an independent-samples t-test, or analyzing only the post-intervention score and ignoring baseline, gives away the design’s statistical power.

Repeated-measures designs with three or more time points use mixed-effects or ANOVA frameworks. Sample size calculation in these designs depends on the assumed correlation structure across time points and is typically conducted using specialized software ( Table 3) rather than a closed-form expression.

### Cluster-randomized educational trials

Educational interventions are often delivered at the level of a classroom, a program, a hospital department, or a cohort, rather than to individual learners. When randomization occurs at the cluster level, sample size calculation must account for the dependence between observations within the same cluster. Treating clustered data as if observations were independent inflates the apparent precision and produces incorrect inference. ^5^

The sample size required for a cluster-randomized trial is the sample size for an individually-randomized trial inflated by the design effect (DE):

DE=1+(m−1)×ICC

where m is the average cluster size, and ICC is the intracluster correlation coefficient, which quantifies the degree to which observations within the same cluster are correlated. ^15^ For HPE interventions delivered at the level of a clinical site or a teaching program, ICC values typically range from 0.01 to 0.10. With an ICC of 0.05 and cluster size m = 20, the design effect is 1 + 19 × 0.05 = 1.95, almost doubling the required sample size compared with an individually-randomized design.

Cluster-randomized trials require careful specification of the number of clusters to randomize, which is typically the more important quantity than the number of individuals within clusters, because increasing cluster size yields diminishing returns once the design effect is fixed; the cluster size, which is often determined by the natural unit (a typical class, a typical residency cohort); the expected ICC for the outcome measured, which should be informed by pilot data or comparable studies, not assumed at zero; and the unit and timing of measurement, which must match the unit of randomization and the time scale of the intervention.

A common error in HPE cluster-randomized trials is to power the study based on the total number of individuals enrolled, ignoring the cluster structure. Studies that randomize four classrooms of 30 students each (total n = 120) and then analyze outcomes as if from 120 independent students are not statistically valid. The effective sample size, accounting for the ICC, may be closer to 40–60. Designs beyond the simple two-arm parallel cluster trial, including three-level designs (learners within clusters within institutions), stepped-wedge designs (in which clusters cross over from control to intervention at staggered time points), and longitudinal cluster designs, are covered in depth elsewhere. ^12^ ^,^ ^13^

### Survey and questionnaire studies

Survey research in HPE serves at least three distinct purposes, and the sample size approach differs for each. Conflating them is a frequent source of error.

Estimation surveys aim to describe the prevalence of a characteristic, attitude, or experience in a defined population. The sample size depends on the target population, the desired precision of the estimate (margin of error), the assumed prevalence of the characteristic of interest, and the chosen confidence level. The standard formula for estimating a population proportion is:

$$n=Z^{2}\times p\left(1-p\right)/E^{2}$$

where Z is the critical value for the chosen confidence level (1.96 for 95% confidence), p is the expected proportion, and E is the acceptable margin of error. ^31^ For p = 0.5 (the most conservative assumption) and E = 0.05 at 95% confidence, the required sample size is approximately 384 completed responses.

When the target population is finite, the finite population correction is applied as follows:

$$n_{\text{adjusted}}=n/\left(1+\left(n-1\right)/N\right)$$

where N is the total population. A survey of an entire residency program with N = 500 trainees requires approximately 218 completed responses to estimate a proportion with 5% margin of error and 95% confidence, rather than 384.

Analytical cross-sectional surveys aim to compare subgroups or to quantify associations rather than to estimate a single prevalence. These are powered using the two-group formulas for means or proportions given above, or using the regression model that will actually be fitted, applied to the comparison of primary interest. A survey powered only to estimate overall prevalence at 5% precision will have much wider precision when stratified by year of training, specialty, or institutional setting, and will usually be underpowered for the comparisons the investigators intend to report. ^14^

Psychometric surveys, in which the instrument’s measurement properties are themselves the object of study, follow the requirements set out in the section on instrument validation below rather than either of the above.

Two further points deserve emphasis. First, the calculated sample size is the number of completed, analysable responses required, not the number of people to invite. Because response rates in HPE surveys are frequently well below 50%, the number of invitations issued must be inflated accordingly: to obtain 384 completed responses at an anticipated response rate of 40%, approximately 960 invitations are required. The anticipated response rate should be based on comparable surveys in comparable populations and stated explicitly alongside the calculation. ^32^ Second, and relatedly, the response rate is not a parameter in the precision calculation itself. Precision is governed by the number of completed responses, the population size and the desired margin of error but it is an essential input to the recruitment plan, and non-response bias remains a threat to validity that a larger number of invitations does not resolve. Studies should report both the target number of completed responses and the number invited, along with the achieved response rate and any assessment of non-response bias. ^32^

### Delphi and consensus studies

The Delphi technique and related consensus methods are widely used in HPE to develop competency frameworks, curricula, assessment blueprints, and quality indicators. Like qualitative research, a Delphi study is not driven by statistical power; the number of participants is an expert panel size rather than a sample drawn to detect an effect. The appropriate panel size depends primarily on the heterogeneity of the expertise required. A systematic review of Delphi studies used to select healthcare quality indicators reported a median panel of 17 members (interquartile range 11 to 31) and noted that more than half of the panels were multi-stakeholder. ^33^

As a practical guide, single-stakeholder or homogeneous panels (for example, a single specialty or discipline) typically function with approximately 10 to 30 experts, whereas multi-stakeholder or heterogeneous panels (for example, clinicians, educators, students, and patients) should be larger, often 30 or more and sometimes up to about 60 to 90, so that each stakeholder group is adequately represented. Panel size should be justified by the diversity of perspectives required and by the need to retain sufficient members across rounds, rather than by a fixed number. A Delphi sample size justification should state the panel composition, the target panel size and its rationale, the number of rounds, the criteria used to define consensus, and the approach to attrition between rounds. ^34^ Table 2 summarises these and other anchors for designs in which a power-based calculation does not apply.

**Table 2.** Commonly cited sample size anchors for designs and methods in which a power-based calculation does not apply.

Method or design	Basis for the number	Commonly cited anchor
Pilot or feasibility study (estimating variance or effect size for a future trial)	Precision of the variance estimate; rule of thumb	≈12 per group (It should be read as an order-of-magnitude anchor rather than a requirement: a pilot with a different purpose, or one estimating a parameter with different precision requirements, will warrant a different number.)
Pilot for questionnaire reliability (Cronbach’s α or test–retest)	Precision of the reliability coefficient	30–50
Delphi (single-stakeholder or homogeneous panel)	Representativeness and stability of consensus within one discipline	~10–30 experts (median ~ 17)
Delphi (multi-stakeholder or heterogeneous panel)	Adequate representation of each stakeholder group across rounds	≈30 or more, commonly up to ≈60–90 (These figures describe what has been done and found workable, not a threshold below which a Delphi study is invalid.)
Qualitative interviews (homogeneous group)	Saturation or information power	9–20 interviews
Cronbach’s α estimation	Precision of the α coefficient	~30
Exploratory factor analysis	Subjects per item; factor loadings and communalities	≥10 per item where communalities are low; substantially fewer may suffice where communalities are high and factors are well determined
Confirmatory factor analysis or structural equation modelling	Model complexity; indicators per latent variable	≈200 or more, scaling with model complexity

The figures above are empirical or precision-based anchors reported in the cited literature and are intended as illustrative starting points for a justified decision. They are not methodological minima, and a smaller or larger sample may be entirely appropriate where the study’s aim, the homogeneity of the sample, and the precision required justify it.

### Pilot and feasibility studies

Pilot and feasibility studies are not powered to detect intervention effects. Their purpose is to estimate parameters needed for a subsequent fully-powered trial: recruitment rates, retention, intervention fidelity, outcome variability, intracluster correlation, or instrument performance. ^28^ Sample size calculation for pilots follows a different logic than sample size calculation for hypothesis-testing studies.

A widely cited rule of thumb of 12 participants per group has been proposed for pilot studies, aimed at estimating effect size and variance for subsequent trials. ^17^ The rule is based on the precision with which the variance of the outcome can be estimated, not on the power to detect an effect. The rule applies when the purpose is parameter estimation for a future definitive trial.

For pilots aimed at testing questionnaire reliability, sample sizes between 30 and 50 are commonly recommended, depending on the number of items and the desired precision of the reliability coefficient (Cronbach’s α or test-retest correlation). For questionnaires with 10 to 20 items targeting a Cronbach’s α of 0.80, approximately 30 participants typically provide stable estimates. ^35^

The defining feature of a properly reported pilot study is that the sample size justification is tied to the pilot’s stated purpose, not to a generic rule of thumb. A pilot designed to estimate recruitment rates may require different numbers than a pilot designed to estimate outcome variance, which in turn differs from a pilot designed to test instrument reliability. A pilot study sample size justification should specify the parameter being estimated, the desired precision, and the source of the rule or formula used.

A common error is to use pilot effect size estimates as the basis for the main trial sample size calculation. Pilot studies are too small to provide stable effect size estimates, because the observed pilot effect is as likely to be an overestimate and the resulting sample size is inversely proportional to its square, using pilot point estimates systematically underestimates the required sample for the definitive trial. ^28^ ^,^ ^29^

### Qualitative research

Qualitative research does not use sample size calculation in the statistical sense, because its purpose is not to estimate population parameters or test hypotheses against a null distribution. The relevant concept is saturation: the point at which additional data collection no longer yields new themes, codes, or insights. ^36^

Saturation as a sample size principle has been criticized for being vague and inconsistently applied. The concept of information power was proposed as an alternative, holding that the sample size required for a qualitative study depends on the study’s aim (broad or narrow), specificity of the sample (specific or sparse), use of established theory (applied or not), quality of dialogue (strong or weak), and analysis strategy (cross-case or case). ^18^ A study with a narrow aim, a specific sample, an established theoretical framework, strong interview dialogue, and case analysis can achieve information power with fewer participants than a study with a broad aim and a sparse sample.

Empirical work on saturation provides practical anchors. For studies aimed at characterizing the perspective of a relatively homogeneous group, the majority of themes typically appear within 12 interviews, and saturation is approached around 12 to 20 interviews. ^36^ A more recent systematic review of empirical saturation studies reported similar findings across diverse qualitative research traditions, with most studies reaching saturation between 9 and 17 interviews. ^37^ These ranges describe what has been observed empirically in particular study contexts; they are not targets to be met, and a study whose aim, sample and analytic strategy differ from those contexts should expect to depart from them.

For HPE qualitative research, a defensible sample size justification combines an information-power assessment with reference to empirical saturation evidence relevant to the study design. Focus group studies have different saturation dynamics than in-depth interviews. A study comparing perspectives across distinct subgroups requires sufficient depth within each subgroup. A study aimed at theory development may require more interviews than a study aimed at descriptive characterisation. The sample size justification should state the principle invoked (saturation, information power, theoretical sampling), the planned monitoring approach during data collection, and the stopping rule.

### Simulation-based education research

Simulation-based education (SBE) is a substantial body of HPE research with its own design features that affect sample size requirements. The systematic review of sample size adequacy in HPE research cited at the start of this guide ^1^ found that the majority of SBE studies were powered to detect only large effects, with median sample sizes in the range of 25 to 30 participants per arm. The systematic review documented that small effect sizes are common in SBE comparison studies, particularly when comparing two active interventions against each other.

The implication is that the field’s typical effect sizes are small relative to the convention, so studies powered on a medium-effect assumption are routinely underpowered for the effects that actually exist. A calculation grounded in published SBE effect size literature (typically *d* = 0.2 to 0.4 for active-versus-active comparisons) yields substantially larger targets than one grounded in Cohen’s medium-effect convention: approximately 175 per group at *d* = 0.3, and approximately 393 per group at *d* = 0.2.

SBE studies also frequently involve multiple outcomes (cognitive, procedural, and attitudinal) and repeated measurements. Multiple-outcome studies require correction for multiple comparisons or a primary outcome specification, and repeated measurements should be analysed using methods appropriate for repeated measures. Sample size calculations should refer to the primary outcome on which the study is powered, rather than multiple secondary outcomes.

A defensible SBE sample size justification cites published effect size estimates from comparable interventions, identifies the primary outcome, specifies the analytical method (paired versus independent, single time point versus repeated), and reports the assumed α, power, and effect size.

### Instrument validation studies

The sample size for instrument validation studies depends on the psychometric property being estimated, and is governed by the precision required for that property rather than by a single participant count.

For internal consistency, approximately 30 participants are commonly cited as providing reasonable precision for an expected Cronbach’s α of approximately 0.80, with more participants required when the expected α is lower or the precision requirements are higher. ^19^ The number follows from the width of the confidence interval the investigator is willing to accept around α, and should be derived for the specific expected value rather than adopted as a fixed figure. Similar sample sizes (30–50 participants) are typically cited for test-retest reliability estimation.

For exploratory factor analysis, the rule of 10 participants per item is widely quoted, but it is the weakest of the available justifications and should be used only where better information is unavailable. Sample size requirements for factor analysis depend far more on the strength of the factor loadings, the communalities of the measured variables, the number of factors extracted, and the ratio of variables to factors than on the number of participants per item. Where communalities are high (≈0.6 and above) and each factor is defined by several strongly loading variables, samples well below the 10:1 rule can recover the factor structure reliably; where communalities are low (≈0.5 or less) and factors are weakly determined, samples of 300 or more may be needed regardless of the number of items. ^38^ Investigators should therefore justify the sample against the expected loading structure and communalities, and should report those expectations.

Confirmatory factor analysis and structural equation modelling typically require larger samples (≥200) and depend on model complexity, the expected effect sizes for parameter estimation, and the number of indicators per latent variable. The COnsensus-based Standards for the selection of health Measurement INstruments (COSMIN) initiative provides structured guidance on the design and appraisal of measurement property studies, including the adequacy of sample size for each property, and is the appropriate reference for HPE instrument validation work. ^39^ ^,^ ^40^ Sample size justifications for validation studies should name the measurement property being assessed, state the precision or model-recovery requirement, and reference COSMIN or comparable measurement-science guidance rather than a generic participant-per-item ratio.

### Designs and approaches beyond the scope of this guide

The designs above account for the majority of published HPE research, but several increasingly common approaches require methods outside the scope of a closed-form guide. They are noted here so that investigators recognise when specialist input is required, with signposts to the appropriate literature.

***Non-inferiority and equivalence studies***

Where the research question is whether a new instructional method is no worse than an established one, the null hypothesis is reversed and the calculation depends on a pre-specified non-inferiority or equivalence margin. This margin must be justified on educational grounds before data collection, and the resulting sample size is often larger than for a superiority design at the same effect size. ^41^ ^,^ ^42^

***Multilevel and mixed-effects designs***

Where learners are nested within tutors, tutors within sites, and measurements within learners, power depends on the variance components at each level as well as on the total sample. Analytical solutions exist for standard cases; ^12^ more complex or unbalanced structures are best handled by simulation. ^43^ ^,^ ^44^

***Longitudinal cohort studies***

Power in cohort designs following learners across training depends on the number and spacing of measurement occasions, the within-person correlation structure, and attrition at each wave, and cannot be reduced to a single-occasion formula. ^16^ ^,^ ^45^

***Adaptive designs***

Trials that pre-specify interim analyses with the option to re-estimate sample size, drop arms, or stop early require a design-stage simulation of the adaptation rules and appropriate control of the Type I error rate across looks. ^46^

***Bayesian approaches***

Bayesian design replaces power with criteria such as the expected width of the posterior interval or the probability of reaching a specified Bayes factor threshold, and requires explicit prior specification; design analysis is typically simulation-based. ^47^

***Mixed-methods studies***

Sample size in mixed-methods research is determined separately for each strand by the logic appropriate to that strand (power or precision for the quantitative component, information power or saturation for the qualitative) and then reconciled against the integration strategy and the point at which the strands meet. ^48^ A single sample size justification covering both strands is not appropriate. In each of these cases, the guidance in this article should be treated as orientation rather than as a substitute for consultation with a statistician or methodologist experienced in the relevant design.

### Software for sample size calculation

Most HPE investigators now calculate sample size in software rather than by hand, and reporting which software was used, with what settings, is part of a reproducible justification. Software does not remove the need for the decisions set out in Steps 1 to 6; it only executes Step 7. The most common errors observed in software-based calculations are choosing the wrong test menu (for example, selecting an independent-samples test for paired data), entering a between-group *d* where the paired *d*_z_ is required, and accepting a default one-sided test without intending one. Table 3 maps the designs covered in this guide to the software routes most commonly available to HPE researchers. Full settings and syntax reproducing every worked example in Table 1 are provided as Extended data, File S1.

**Table 3.** Software routes for sample size calculation by study design.

Study design	G*Power 3.1	R	Commercial (PASS, Stata, SAS)
Two-group means	t tests → Means: Difference between two independent means (two groups)	pwr::pwr.t.test	PASS: Two-Sample T-Test; Stata: power twomeans; SAS: PROC POWER TWOSAMPLEMEANS
Two-group proportions	z tests → Proportions: Difference between two independent proportions	pwr::pwr.2p.test	PASS: Two Proportions; Stata: power twoproportions; SAS: PROC POWER TWOSAMPLEFREQ
Paired (pre–post)	t tests → Means: Difference between two dependent means (matched pairs)	pwr::pwr.t.test (type="paired")	PASS: Paired T-Test; Stata: power pairedmeans
Repeated measures (≥3 occasions)	F tests → ANOVA: Repeated measures, within factors	WebPower::wp.rmanova; simr for mixed models	PASS: Repeated Measures ANOVA; Stata: power repeated
Cluster-randomized	Not directly supported; apply design effect to the individually-randomized result	clusterPower; simr::powerSim	PASS: Cluster-Randomized Designs; Stata: power twomeans, cluster
Survey (proportion estimation)	Not applicable (precision, not power)	presize::prec_prop; closed-form	PASS: Confidence Intervals for One Proportion; Stata: ciwidth onemean
Correlation	Exact → Correlation: Bivariate normal model	pwr::pwr.r.test	PASS: Correlations; Stata: power onecorrelation
Regression (multiple predictors)	F tests → Linear multiple regression: Fixed model, R ^2^ increase	pwr::pwr.f2.test	PASS: Multiple Regression
Logistic regression	z tests → Logistic regression	WebPower::wp.logistic	PASS: Logistic Regression; SAS: PROC POWER LOGISTIC
Multilevel/mixed-effects	Not supported	simr (simulation-based)	PASS: Mixed Models; Stata: power with simulation
Reliability (Cronbach’s α)	Not supported	psychometric::alpha.CI; closed-form	PASS: Cronbach’s Alpha
Factor analysis (EFA/CFA/SEM)	Not supported	semPower; lavaan with simulation	Mplus Monte Carlo facility
Precision-based planning (any)	Not directly supported	presize	PASS: Confidence Intervals modules; Stata: ciwidth

### Adjusting for expected attrition and missing data

Most HPE studies experience some loss of participants between recruitment and analysis. The sample size calculated from any of the formulas in this guide represents the number of participants required *at analysis*, not at recruitment. To account for expected attrition, the calculated sample size N should be inflated to a recruitment target N _d_:

$$N_{d}=N/\left(1-d\right)$$

where d is the expected proportion lost to drop-out or non-compliance. For a study requiring N = 100 participants at analysis with an expected drop-out rate of 20% (d = 0.20), the recruitment target becomes N _d_ = 100/(1 − 0.20) = 125. The choice of d should be informed by the attrition rates observed in comparable studies, the length and intensity of the intervention, and population characteristics. Sample size justification should report both the calculated N and the inflated recruitment target N _d_, along with the source of the assumed attrition rate. ^30^

Inflation protects statistical power; it does not protect validity. Three further considerations apply.

***Differential attrition***

Where drop-out differs systematically between arms, the groups that remain are no longer comparable, and the resulting bias is not corrected by recruiting more participants. Anticipated differential attrition should be addressed at the design stage through the retention strategy and, where it is expected, the analysis plan should specify how it will be examined. Attrition by arm and the reasons for it should be reported.

***Missing data mechanisms***

The appropriate handling of missing outcomes depends on why the data are missing. Where missingness is unrelated to any observed or unobserved variable (missing completely at random), a complete-case analysis is unbiased but inefficient. Where missingness depends on observed variables (missing at random), complete-case analysis is generally biased and principled methods are required. Where missingness depends on the unobserved outcome itself (missing not at random), no method recovers the truth without untestable assumptions, and sensitivity analysis is the appropriate response. ^49^

***Multiple imputation and analysis-stage implications***

Multiple imputation, mixed-effects models using all available observations, and inverse-probability weighting each make different assumptions and each have implications for the effective sample size. Where multiple imputation is planned, the analysis plan should state the imputation model, the variables included in it, and the number of imputations, and the study should not rely on imputation to substitute for a retention strategy. ^49^ The sample size justification should state which approach to missing data is planned, because a study analysed by complete cases requires a larger recruitment target than one analysed with all available data.

### Reporting sample size justification

A methods section should report sample size justification in sufficient detail that an independent reader can reproduce the calculation. The Statistical Analyses and Methods in the Published Literature (SAMPL) guidelines recommend that sample size reporting include the primary outcome on which the study is powered, the analytical method, the assumed effect size and its source, the chosen α and power, any adjustments for design effect or correlation, and the calculated required sample size. ^20^ For pilot and qualitative studies, the analogous information includes the parameter being estimated or the saturation or information-power principle applied the source for any rule of thumb invoked, and the planned monitoring approach during data collection. Table 4 sets out these requirements as a checklist.

**Table 4.** Checklist for reporting a sample size justification.

#	Item	Report
1	Primary outcome	The single outcome on which the study is powered or sized, and its measurement scale
2	Study design and unit of analysis	The design, and the unit at which observations are treated as independent
3	Analytical model	The specific test or model the calculation corresponds to
4	Aim of the calculation	Whether the study is sized to detect an effect (power) or to estimate one (precision)
5	Effect size or target precision	The assumed effect size or target interval width, with its numerical value
6	Source of the effect size	The specific study, meta-analysis, or judgment the value comes from, and why its comparator and population match
7	α, power, and test direction	The significance level, the power, and whether the test is one- or two-sided
8	Allocation ratio	The planned ratio between arms, stated even when 1:1
9	Design adjustments ^†^	Design effect and ICC (cluster designs); within-subject correlation (paired/repeated); finite population correction (surveys); multiplicity adjustment where planned
10	Calculated sample size at analysis	The resulting N, per group and in total
11	Attrition or non-response inflation	The assumed rate, its source, and the resulting recruitment target; for surveys, the number of invitations required
12	Missing data approach	The planned analysis-stage handling of missing outcomes
13	Software	The program, version, and procedure or menu path used
14	Non-power designs ^†^	For pilots: the parameter estimated and precision sought. For qualitative: the principle invoked and the stopping rule. For Delphi: panel composition, target size, rounds, and consensus criteria
15	Achieved sample	The final analysed sample, the reason for any shortfall, and its implications for the interpretation of the results

^†^ Items 1–10 apply to all designs; items marked with a dagger apply to the designs indicated.

Sample size justifications should also report what was actually achieved. Studies that fail to reach the planned sample size should report the final sample, the reason for the shortfall, and the implications for power. Studies that exceed the planned sample size should report the final sample and the rationale without retrospectively re-powering the analysis.

Common reporting deficiencies that this guide aims to help authors avoid include: stating a sample size without justification; citing a software output without parameters; citing a rule of thumb without naming the rule or its source; failing to specify the unit of analysis; calculating power on a secondary outcome when the analysis is reported on a primary outcome; and reporting power calculated retrospectively from observed data.

Reviewers and editors evaluating sample size justifications should check that the design, the analytical method, the assumed effect size, the chosen α and power, the unit of analysis, and the final sample size are all internally consistent. Inconsistency among these components is a common signal of sample size determination that was not done before data collection but was reconstructed afterward.

### Use cases

This guide brings established sample size procedures together into one workflow organized by study design rather than proposing a new estimator, so formal validation through simulation or empirical accuracy testing does not apply. The formulas and rules it uses are already validated in the statistical literature cited throughout, including Cohen’s effect size conventions, the cluster design effect, the finite population correction, the information power approach, and COSMIN guidance. Each design section shows them applied through a worked example set in HPE practice, and the logic of the guide extends the authors’ earlier work on sample size in educational research. ^7^

The workflow is meant for use at the design stage: investigators identify the design and unit of analysis, choose the matching model, anchor the effect size to a source matched to the comparator and to educational importance, decide whether the study is sized to detect or to estimate, justify α and power, adjust for clustering or repeated measures, apply the formula in the software of their choice, inflate for attrition and non-response, and report against the checklist in Table 4. Reviewers and editors can follow the same sequence as an appraisal checklist. Its value is to reduce the two documented failure modes, silent underpowering and post hoc justification, by giving the field one reference matched to the design. Peer review will be most useful in testing the worked examples and the effect size ranges suggested for each comparator class.

### Limitations

Several limitations should be borne in mind when using this guide.

It is a practical methodological guide, not a systematic review or a consensus statement. As set out in the Methods, the literature was searched purposively rather than systematically, selection was not duplicated independently, and the included sources were not formally appraised; other authors synthesising the same field might reasonably emphasise different sources.

The numerical anchors reproduced in Tables 1 and 2 are illustrative rather than prescriptive. Each derives from a specific context and set of assumptions, and applying any of them outside that context particularly the pilot, saturation, and factor-analysis figures without considering the study’s own aim and precision requirements would repeat the error the guide is intended to correct.

The closed-form formulas presented here apply to the simple, balanced, single-outcome cases that account for most HPE research. They do not extend to the designs listed in the section on designs beyond the scope of this guide, nor to unbalanced or three-level cluster structures, complex missing-data patterns, or models with several correlated outcomes.

Finally, no guide substitutes for methodological expertise. Investigators planning studies with complex designs, unusual outcome structures, or high-stakes implications should involve a statistician or methodologist at the design stage, not after data collection. The purpose of this guide is to make that conversation more productive by clarifying what the investigator must decide before it begins.

## Conclusions

Sample size determination in health professions education research is design-specific rather than formula-specific. The required sample size for a study follows from the research question, study design, expected effect size or target precision, chosen significance level and power, unit of analysis, and analytical approach. Each of these inputs is design-specific, and changing any of them changes the required sample size. The widespread search for a universal formula reflects a methodological misunderstanding that this guide has aimed to dispel.

The practical implication for HPE investigators is that sample size belongs at the design stage of a study, integrated with the choice of research question, study design, and analytical strategy, and not added as a post-hoc justification. The practical implication for reviewers and editors is that sample size justifications should be evaluated against the design they support, and not against generic conventions imported from other disciplines.

Two developments are likely to change how this work is done. The first is the continued displacement of closed-form calculation by simulation-based design analysis, which imposes no restriction on the complexity of the model and can accommodate the nested, longitudinal, and multi-outcome structures that characterise educational research but defeat analytical formulas. ^43^ ^,^ ^44^ As accessible implementations mature, simulation is likely to become the default rather than the specialist route for HPE designs of any complexity. The second is the growing use of large language models and other artificial intelligence tools to assist with study design, including sample size planning. These tools can generate plausible-looking calculations without the design reasoning that makes a calculation defensible, and they reproduce the same conventions, uncritically applied, that this guide argues against.

## AI disclosure

The authors used Claude (Anthropic; Claude Opus 4.x) to assist with language editing. No AI tool was used to generate, select, or verify the statistical content, formulas, or numerical examples. All AI-assisted output was critically reviewed, verified, and edited by the authors, who take full responsibility for the originality, accuracy, and integrity of the content.

## Data Availability

No primary data are associated with this article. Zenodo: *Extended data for: Sample size determination across study designs in health professions education research: a methodology guide.* https://doi.org/10.5281/zenodo.21929103 This project contains the following extended data:
• File S1 — Software settings and syntax to reproduce every worked example in Table 1 (G*Power 3.1 menu paths and input parameters; R script using the **pwr** package; expected output for each example; a reconciliation of closed-form and software values; and a prose template for reporting a sample size justification). Data are available under the terms of the Creative Commons Attribution 4.0 International license (CC-BY 4.0). Not applicable. This article is a methodological guide and does not report primary empirical research; no reporting-guideline checklist applies.
