# Supplementary material for: Sample size determination across study designs in health professions education research: a methodology guide
Source: F1000Res. 2026 Sep 3;15:1147. Originally published 2026 Jul 13. [Version 2] doi: 10.12688/f1000research.185853.2 (PMC13562950; doi:10.12688/f1000research.185853.2)
Supplement: Peer review history

# Reviewer report: Reviewer response for version 2

**DOI:** 10.5256/f1000research.208637.r517847

**Published:** 2026-09-29

**Authors:** Dipro Mondal

**Affiliations:** 1 Tilburg University, Tilburg, The Netherlands

The manuscript presents a cohesive guide for health professionals education to sample size determination and planning. The paper does not really propose a new or unique method for sample size determination but does a good job in consolidating what methodology there is on sample size determination and how to approach the sample size calculation step-by-step. The manuscript also does an impressive job in carefully addressing missing data mechanisms and its consequence on sample size. Although the methods described in the paper are generally easy to follow, some parts of the general framework needs clarification, particularly regarding the interpretation of sample size and the conclusions that can be drawn from a given calculation.

 1. **Clearer explanation of why sample size matters**

 The Introduction motivates the sample size determination in terms of statistical power. This is indeed correct, but a more fundamental connection between sample size, variability and sampling uncertainty is lacking. An estimate calculated from a sample is itself subject to sampling variation, thereby affecting the standard error, confidence interval (width), and statistical power. The paper partly addresses this later through precision-based planning, using the width of the confidence interval. However, the connection between these concepts is lacking, and I think this should already be mentioned in the Introductory part. I suggest connecting the ideas explicitly so that power and precision are presented as consequences of the same underlying sampling uncertainty. In the discussion, it might also be useful to state that increasing sample size reduces sampling variability but does not remove systematic problems such as selection bias, or model misspecification.

 2. **Sampling mechanism is not generally discussed**

 The manuscript mentions 'sampling' in several places and in different contexts. The authors mainly focus on the unit of analysis and its relation to the unit of randomization. However, the way in which participants are sampled or recruited can also affect the interpretation of the results. A convenience sample, for example, may differ systematically from a probability based sample drawn from the target population, even when both have the same sample size. This inturn affects the generalizability of the results of a study done after calculating a sample size using proper methods. A short discussion on this may help clarify the limitations of sample size calculations in general.

 3. **Monte Carlo Sample Size as a general framework**

 The manuscript already recognizes that a sample size determination for complex statistical methodologies is not always possible, in which case simulations might help determining sample size. I think Monte Carlo simulation should be introduced more directly rather than an alternative solution when a closed form solution is unavailable. Even when a closed form solution is available, Monte Carlo simulations can be used for checking whether the calculated sample size provides adequate statistical properties Muthén & Muthén (2002). However, it also needs to be stated that Monte Carlo simulations are not a failproof option. Morris et al. (2019) emphasize that simulation studies should themselves be carefully designed, evaluated and Monte Carlo errors carefully reported.

 Refer (1)

 Refer (2)

Is the rationale for developing the new method (or application) clearly explained?

Yes

Is the description of the method technically sound?

Partly

Are the conclusions about the method and its performance adequately supported by the findings presented in the article?

Partly

If any results are presented, are all the source data underlying the results available to ensure full reproducibility?

No source data required

Are sufficient details provided to allow replication of the method development and its use by others?

Yes

Reviewer Expertise:

Optimal Design, Sample Size determination, Uncertainty Quantification

I confirm that I have read this submission and believe that I have an appropriate level of expertise to confirm that it is of an acceptable scientific standard, however I have significant reservations, as outlined above.

## References

[1] : How to Use a Monte Carlo Study to Decide on Sample Size and Determine Power. *Structural Equation Modeling: A Multidisciplinary Journal* .2002;9(4) : 10.1207/S15328007SEM0904_8599-620 10.1207/S15328007SEM0904_8

[2] : Using simulation studies to evaluate statistical methods. *Statistics in Medicine* .2019;38(11) : 10.1002/sim.80862074-2102 10.1002/sim.8086 PMC649216430652356

# Reviewer report: Reviewer response for version 2

**DOI:** 10.5256/f1000research.208637.r517506

**Published:** 2026-09-16

**Authors:** Sushma Prabhath

**Affiliations:** 1 Manipal Academy of Higher Education, Manipal, Karnataka, India

Thank you to the authors for the careful and comprehensive revision of this manuscript. I have reviewed the revised version alongside the concerns raised during the previous round of peer review and am satisfied that the substantive issues have been adequately addressed.

 The revised manuscript is considerably strengthened. In particular, the addition of a clear description of how the methodology guide was developed improves the transparency of the work. The authors appropriately identify the article as a structured narrative synthesis rather than a systematic review or formal consensus document and clearly describe the literature sources, selection principles, and approach used when recommendations differed.

 I also appreciate the more cautious treatment of numerical sample-size recommendations. The revised manuscript appropriately presents these as illustrative or commonly cited anchors rather than universal methodological thresholds. This is particularly important for pilot studies, qualitative research, Delphi studies, and instrument validation, where rigid numerical rules may otherwise be misinterpreted by less experienced researchers.

 The additional discussion of precision-based sample-size planning, educational versus statistical significance, complex study designs, missing data and attrition, and software-based implementation has substantially improved the methodological breadth and practical relevance of the article. The distinction between descriptive/estimation surveys, analytical cross-sectional studies, and psychometric studies is also helpful.

 The practical usability of the guide has been considerably enhanced by the revised Table 1, the nine-step workflow, the decision tree, the software guidance, and particularly the reporting checklist. These additions make the article useful not only to researchers planning HPE studies but also to postgraduate students, supervisors, peer reviewers, and journal editors.

 The authors have also improved reproducibility by explicitly stating the assumptions underlying the worked examples, reconciling differences between closed-form calculations and software-derived estimates, and providing software settings and syntax as extended data.

 Overall, I consider the revised manuscript to be a useful, well-structured, transparent, and practically relevant methodological resource for health professions education research. The authors have responded constructively and comprehensively to the issues raised during peer review, and I do not have any further substantive concerns requiring revision.

**I am satisfied with the revised manuscript and recommend approval in its present form.**

Is the rationale for developing the new method (or application) clearly explained?

Yes

Is the description of the method technically sound?

Partly

Are the conclusions about the method and its performance adequately supported by the findings presented in the article?

Yes

If any results are presented, are all the source data underlying the results available to ensure full reproducibility?

Yes

Are sufficient details provided to allow replication of the method development and its use by others?

Yes

Reviewer Expertise:

Medical education, Interprofessional education, Health professions education, Humanities, Bioethics

I confirm that I have read this submission and believe that I have an appropriate level of expertise to confirm that it is of an acceptable scientific standard.

# Reviewer report: Reviewer response for version 2

**DOI:** 10.5256/f1000research.208637.r517508

**Published:** 2026-09-10

**Authors:** Vivek Harsukhbhai Ramanandi

**Affiliations:** 1 Faculty of Physiotherapy, Marwadi University, Rajkot, Rajkot, India

Dear Authors!

 You have commendably addressed all major and minor comments from the review round, point by point, in your response and the revised manuscript. I think the article can be satisfactorily indexed without further changes.

 Congratulations!

Is the rationale for developing the new method (or application) clearly explained?

Yes

Is the description of the method technically sound?

Partly

Are the conclusions about the method and its performance adequately supported by the findings presented in the article?

Yes

If any results are presented, are all the source data underlying the results available to ensure full reproducibility?

No source data required

Are sufficient details provided to allow replication of the method development and its use by others?

Partly

Reviewer Expertise:

Neurological physiotherapy, Musculoskeletal disorders, Health professions education research

I confirm that I have read this submission and believe that I have an appropriate level of expertise to confirm that it is of an acceptable scientific standard.

# Reviewer report: Reviewer response for version 1

**DOI:** 10.5256/f1000research.205135.r504181

**Published:** 2026-08-07

**Authors:** Manjush Karthika

**Affiliations:** 1 Liwa University, Abu Dhabi, United Arab Emirates

**Thank you for giving me the opportunity to review this manuscript.**

 This is a useful and practical paper. Sample size calculation is often difficult for health professions education researchers, particularly when different study designs are used. The authors have brought the main approaches together clearly. The tables and worked examples will be helpful for postgraduate students and early-career researchers. I have a few comments which may improve the clarity and practical use of the paper.

1. **Abstract and the section on effect size**

The Abstract states that the effect size may be taken from a pilot study. Later, in the section on pilot and feasibility studies, the authors correctly mention that an effect size from a small pilot can be unstable. Please make these two statements consistent. I suggest mentioning that pilot work can help estimate variability, recruitment and attrition, while the expected difference should mainly be based on previous evidence and educational importance.

1. **Table 1 and the worked examples**

Please add the main assumptions used in each example, either below Table 1 or with the individual examples. This should include alpha, power, whether the test is one- or two-sided, allocation ratio and whether attrition was added. When a software package or online calculator was used, please name it. This will make it easier for readers to repeat the calculation.

1. **Survey section and limitations**
  Please distinguish between the required number of completed responses and the number of participants who need to be invited based on the anticipated response rate. In addition, a brief limitations section acknowledging that this is a practical methodological guide and that complex study designs may require specialised statistical support would strengthen the manuscript.

Minor point: Please check the manuscript once for small language, abbreviation and equation-formatting issues, including “New York” instead of “Newyork”.

1. **Reporting checklist **

Please consider including a brief sample-size reporting checklist (or boxed summary) listing the minimum items authors should report (primary outcome, effect size source, α, power, allocation ratio, attrition adjustment, software used, and final recruitment target). This would improve the paper's practical utility.

1. **Software examples**

Where software is recommended (e.g., GPower, PASS, R), it may be useful to provide one worked example with the exact software settings or syntax in supplementary material.

1. **Visual workflow**

A simple flowchart summarising the nine-step sample-size determination process would help readers quickly identify the appropriate pathway for different study designs.

 Overall, this is a timely and valuable methodological guide that addresses an important gap in health professions education research. Addressing the above points would further enhance its clarity, reproducibility, and usefulness for researchers, educators, and journal reviewers.

Is the rationale for developing the new method (or application) clearly explained?

Yes

Is the description of the method technically sound?

Yes

Are the conclusions about the method and its performance adequately supported by the findings presented in the article?

Yes

If any results are presented, are all the source data underlying the results available to ensure full reproducibility?

Yes

Are sufficient details provided to allow replication of the method development and its use by others?

Yes

Reviewer Expertise:

NA

I confirm that I have read this submission and believe that I have an appropriate level of expertise to confirm that it is of an acceptable scientific standard, however I have significant reservations, as outlined above.

# Response

**Published:** 2026-08-14

**Authors:** Anusha Divvi

**Affiliations:** Sri Balaji Vidyapeeth University, Puducherry, Puducherry, India

We thank the reviewer for a report focused on practical usability, which prompted two of the changes we think most improve the guide.

**1. Inconsistency between the abstract and the effect size section regarding pilot studies.**

 Confirmed, and a real internal contradiction. Resolved in the direction you suggest, in three places. The abstract now reads: "we emphasise that the expected difference should be anchored to previous evidence and to educational importance, with pilot work used to estimate variability, recruitment and attrition rather than the effect size itself." Step 4 no longer presents pilots and published literature as parallel options, stating that pilots "are too small to provide stable effect-size point estimates; their point estimates should not be used directly to power a definitive trial." The pilot section now gives the reason Version 1 asserted without explaining: "because the observed pilot effect is as likely to be an overestimate as an underestimate and the resulting sample size is inversely proportional to its square, using pilot point estimates systematically underestimates the required sample." Figure 1, Step 4 also carries "Not a pilot point estimate."

**2. Table 1 and the worked examples — state the assumptions and name the software.**

 A standing assumptions note now appears beneath Table 1: "α = 0.05, two-sided; power = 0.80; 1:1 allocation; complete data with no attrition inflation applied; no adjustment for multiplicity; simple random sampling for survey rows; and, for the two-group means rows, equal variances in the two groups." A second note names the software and reports the small differences between closed-form and software results rather than leaving a reader who runs G*Power to discover them unexplained; the full reconciliation is given in our response to Reviewer 2, technical point 3. Table 1 also carries a *Key assumption and common pitfall* column. Full settings and syntax are in *Extended data*, File S1 ( https://doi.org/10.5281/zenodo.21929103).

**3a. Survey section — completed responses versus invitations.**

 A real gap, and correcting it required softening an overstatement. Version 1 said flatly that "response rate is a practical consideration in recruitment, not a parameter in sample size calculation," which is true of the precision calculation but not of the recruitment plan, and as written it discouraged the calculation you are asking for. Version 2 states that "the calculated sample size is the number of *completed, analysable responses* required, not the number of people to invite," with the worked figure: "to obtain 384 completed responses at an anticipated response rate of 40%, approximately 960 invitations are required." Step 8 is retitled "Inflate for expected attrition and, in surveys, for non-response," and item 11 of the checklist asks for the number of invitations.

**3b. Limitations section.**

 A new *Limitations* section has been added before Conclusions, covering: that this is a practical guide rather than a systematic review or consensus statement; that the numerical anchors are illustrative; that the closed-form formulas apply to simple, balanced, single-outcome cases only; and, as you ask, that complex designs require specialised support "no guide substitutes for methodological expertise. Investigators planning studies with complex designs, unusual outcome structures, or high-stakes implications should involve a statistician or methodologist at the design stage, not after data collection."

**3c. Language, abbreviations, equation formatting, and "New York".**

 The affiliation is corrected. Notation is standardised. Every abbreviation is defined at first use, and HPE, SBE, COSMIN, SAMPL, ICC, DE, EFA, CFA, SEM and SMD are also defined in the figure footnotes so the figures stand alone. All display equations have been reset on their own lines, including the unequal-allocation formula, which contained a genuine error.

**4. Reporting checklist.**

 New Table 4 provides fifteen items. Every element you specified is included: primary outcome (1), effect size source (6), α and power (7), allocation ratio (8), attrition adjustment and recruitment target (11), and software (13) with six further items the three reports collectively implied: unit of analysis (2), analytical model (3), detect-versus-estimate (4), test direction (7), missing data approach (12), and achieved sample (15). A prose reporting template is given at the end of File S1.

**5. Software examples with exact settings or syntax in supplementary material.**

 Addressed in full; File S1 contains nine worked examples with G*Power field lists, runnable R syntax and expected output, including the simr route for cluster designs, which G*Power does not support. It also flags the specific input errors that produce wrong answers in G*Power, notably entering a between-group *d* where *d*z is required.

**6. Visual workflow.**

 Two figures created; Version 1 had none. Figure 1 presents the nine-step workflow grouped into four labelled phases. Figure 2 is a decision tree routing the reader from study aim through the unit of analysis to the applicable method, with a common tail for attrition inflation and reporting and a flagged branch for designs needing specialist input. We combined the decision tree and design-to-formula algorithm deliberately, since presented separately they would have duplicated the same branching structure.

# Reviewer report: Reviewer response for version 1

**DOI:** 10.5256/f1000research.205135.r504186

**Published:** 2026-08-06

**Authors:** Vivek Harsukhbhai Ramanandi

**Affiliations:** 1 Faculty of Physiotherapy, Marwadi University, Rajkot, Rajkot, India

This is a thoughtful and timely article that consolidates sample-size guidance for various HPE study designs into a practical reference, addressing a clear need in the field.

 The writing is clear, the organization by study design is logical, and Table 1 will likely serve as a valuable quick-reference tool.

 However, there are a few gaps that can be addressed:

 1. The article does not describe the literature search process or how recommendations were selected, which is essential for a methodology guide.

 2. Some numeric benchmarks (such as 12 participants per group for pilots, 30 for Cronbach's alpha, and 10 subjects per item for EFA) are presented more definitively than the supporting literature warrants. These should be framed as guidelines rather than strict rules.

 3. The guide would also benefit from a brief section on confidence-interval or precision-based planning, practical software recommendations (such as G*Power, R, or PASS), and discussion of additional designs, including mixed-methods, non-inferiority, and multilevel models.

 There are also some technical issues.

 1. Notations are inconsistent (for example, α/β versus alpha/beta, and Cronbach's alpha versus α) and should be standardized.

 2. The unequal-allocation formula appears to have a formatting error that should be reviewed in both the HTML and PDF versions.

 3. Including software syntax for the examples in Table 1 would enhance reproducibility, which the authors encourage.

 A few writing issues remain, including tense inconsistencies when describing formula outputs, a near-duplicate between the Objectives section and the Methods paragraph in the abstract, a typo in one author affiliation ("Newyork"), and a missing space in reference 6.

 Overall, these issues do not affect the scientific validity or usefulness of the guide but addressing them would make it a stronger and more user-friendly reference.

Is the rationale for developing the new method (or application) clearly explained?

Yes

Is the description of the method technically sound?

Partly

Are the conclusions about the method and its performance adequately supported by the findings presented in the article?

Yes

If any results are presented, are all the source data underlying the results available to ensure full reproducibility?

No source data required

Are sufficient details provided to allow replication of the method development and its use by others?

Partly

Reviewer Expertise:

Neurological physiotherapy, Musculoskeletal disorders, Health professions education research

I confirm that I have read this submission and believe that I have an appropriate level of expertise to confirm that it is of an acceptable scientific standard, however I have significant reservations, as outlined above.

# Response

**Published:** 2026-08-14

**Authors:** Anusha Divvi

**Affiliations:** Sri Balaji Vidyapeeth University, Puducherry, Puducherry, India

We thank the reviewer for a concise report that identified several concrete technical errors we had missed.

**Major 1 — The article does not describe the literature search process or how recommendations were selected.**

 A new section, *Methods: how this guide was developed*, has been added between Objectives and Main body. It states the three literature streams drawn on; the databases (MEDLINE/PubMed and Google Scholar), the search terms, and the use of forward and backward citation chaining; three explicit inclusion criteria; and an acknowledgement that no formal quality appraisal and no dual independent selection were performed. It closes by stating that the guide "has not been subjected to a Delphi or other formal consensus process, and it does not carry the authority of a consensus statement."

**Major 2 — Some numeric benchmarks are presented more definitively than the supporting literature warrants.**

 Accepted in full; this was the most consistent point across all three reports. It is addressed at four levels. The abstract Conclusions now state that "the numerical benchmarks reproduced here are illustrative anchors rather than methodological minima." Table 2 has been retitled *"Commonly cited sample size anchors for designs and methods in which a power-based calculation does not apply"*, its column heading changed from "Minimum or typical sample" to "Commonly cited anchor", and a standing caveat added beneath it. Each benchmark you named has been individually softened: the 12-per-group pilot rule is now "an order-of-magnitude anchor rather than a requirement"; the 30 for Cronbach's α is "commonly cited as providing reasonable precision" and should be "derived for the specific expected value rather than adopted as a fixed figure"; the 10:1 EFA rule is "the weakest of the available justifications"; and the 9–20 interview range now carries "these ranges describe what has been observed empirically in particular study contexts; they are not targets to be met." The new Limitations section states that applying these anchors outside their originating context "would repeat the error the guide is intended to correct."

**Major 3 — Confidence-interval or precision-based planning; software recommendations; additional designs.**

 Three separate additions: a new *Planning for precision rather than power* subsection with two worked examples and a Table 1 row; a new *Software for sample size calculation* section with Table 3; and a new *Designs and approaches beyond the scope of this guide* section.

**Technical 1 — Notation is inconsistent.**

 Standardised throughout: α for the significance level in prose and formulas; β with power as 1 − β; Cronbach's α; italic *d* and *d*z; Z₁₋α/₂ and Z₁₋β in that form in every formula and in Table 1; σ and σ²_d; *r* for within-subject correlation and ICC for intracluster correlation; m for cluster size; N and N_d for the analysis sample and recruitment target. Version 1 used "alpha" in prose in several places and mixed Z-subscript styles between Table 1 and the body text; both are now uniform.

**Technical 2 — The unequal-allocation formula appears to have a formatting error.**

 Confirmed, and this was a genuine error rather than only a rendering artefact. The superscript 2 had detached from Δ and attached to a stray comma, so the denominator read as Δ rather than Δ². The formula is now a display equation on its own line, and the definition of n₂ — omitted entirely in Version 1 — has been added: n₁ = (Z₁₋α/₂ + Z₁₋β)² × σ² × (1 + 1/r)/Δ², with n₂ = r × n₁, the total minimised at r = 1. All other display equations have been checked. We would ask the production team to confirm the rendering in both HTML and PDF, as you suggest.

**Technical 3 — Including software syntax for the examples in Table 1 would enhance reproducibility.**

*Extended data*, File S1, has been deposited on Zenodo ( https://doi.org/10.5281/zenodo.21929103, CC-BY 4.0) and provides, for each of nine worked examples, the standing assumptions, the exact G*Power 3.1 test family and input fields, runnable R syntax using the pwr package, and the expected output. It covers cluster designs via simr, precision-based planning, reliability, and factor analysis via semPower.

 Working through this revealed that the closed-form values in Table 1 and the software values are not always identical: for two independent means, 63 per group against 64 by exact noncentral *t* at *d* = 0.5, 175 against 176 at *d* = 0.3, 393 against 394 at *d* = 0.2; for two proportions, 167 against 170 using Cohen's arcsine-transformed *h* = 0.305 in pwr.2p.test, where 167 delivers 79.5% rather than 80.0% power; 34 by both routes for the paired example; and 384 for the survey figure by conventional rounding of 384.16, where strict rounding gives 385.

 We have retained the closed-form values in Table 1, because they are what the formulas printed alongside them produce, and reported the discrepancies in an expanded note beneath the table rather than silently substituting software output for formula output: "the differences are small but real, and are reported here rather than concealed, because a guide that asks investigators to report their software must show what difference the software makes."

**Writing 1 — Tense inconsistencies.**

 All formula outputs are now in the present tense. The five instances corrected were: "sharply increased to approximately 393" → "rises sharply to"; "the required sample size was approximately 384 participants" → "is approximately 384 completed responses"; "sample sizes between 30 and 50 were recommended" → "are commonly recommended"; "was proposed for pilot studies, which was aimed at estimating" → "has been proposed for pilot studies aimed at estimating"; and "The concept of information power was proposed, which holds" → "was proposed as an alternative, holding".

**Writing 2 — Near-duplicate between Objectives and the abstract Methods paragraph.**

 Objectives has been rewritten entirely. Version 1 restated the abstract's Methods paragraph almost verbatim. Objectives now states one objective and three specific aims, with no overlap; the abstract Methods paragraph has correspondingly been rewritten to describe the development method, which was the substance of your first major point.

**Writing 3 — "Newyork".**

 Corrected to "New York" in affiliation 4, and in the submission system author metadata.

**Writing 4 — Missing space in reference 6.**

 Corrected; the missing journal title has also been added. The list has been rechecked, renumbered in order of first appearance, and standardised for volume, issue, page range and DOI format.

# Reviewer report: Reviewer response for version 1

**DOI:** 10.5256/f1000research.205135.r504180

**Published:** 2026-07-28

**Authors:** Sushma Prabhath

**Affiliations:** 1 Manipal Academy of Higher Education, Manipal, Karnataka, India

**Reviewer Report**

 This manuscript addresses an important and frequently misunderstood aspect of health professions education (HPE) research: sample size determination across different study designs. The authors should be commended for attempting to consolidate dispersed methodological guidance into a single practical resource tailored to HPE researchers. The manuscript is well structured, clearly written, and likely to be useful for novice researchers, reviewers, and educators.

 However, although presented as a "methodology guide", the manuscript is largely a narrative synthesis of existing recommendations rather than a formal methodological framework. Some recommendations are presented too definitively despite being based on rules of thumb rather than universally accepted methodological standards. In addition, several important methodological nuances are simplified or omitted. Addressing these issues would substantially improve the scientific rigor and long-term usefulness of the guide.

**Major comments**

**1. Clarify the methodology used to develop this guide**

 The manuscript describes itself as a methodology guide, yet it is not clear how the guidance was developed.

 The authors cite statistical textbooks, methodological papers and HPE literature, but there is no description of:

- how references were identified,
- whether literature was systematically searched,
- whether guidance was selected using predefined criteria,
- how conflicting recommendations were reconciled.

Consequently, readers cannot determine whether this represents:

- an expert narrative review,
- a consensus document,
- or a structured methodological synthesis.

**Recommendation**

 Include a brief Methods section describing:

- literature sources
- search strategy (if applicable)
- selection approach
- rationale for included recommendations

This would considerably strengthen the credibility of the guide.

**2. Some recommendations are presented too prescriptively**

 Throughout the manuscript, certain sample size recommendations are written as if they are universally accepted.

 Examples include:

- 12 participants per group for pilot studies
- 30 participants for Cronbach alpha
- 10 participants per questionnaire item
- ≥200 for CFA
- 9–20 interviews for qualitative research

These are useful practical benchmarks but remain context-dependent.

 Modern methodological literature increasingly discourages rigid numerical thresholds.

 The manuscript should consistently emphasise that these values are illustrative guidance rather than minimum methodological requirements.

**3. More discussion is needed regarding software implementation**

 The guide frequently refers to formulas but provides relatively little practical guidance regarding modern software.

 Most HPE researchers now calculate sample size using software rather than manual equations.

 Consider briefly discussing:

- G*Power
- R packages (pwr, simr)
- PASS
- Stata
- SAS

A table matching study designs with commonly used software would substantially improve practical usability.

**4. Limited discussion of complex contemporary HPE study designs**

 The manuscript appropriately covers common designs but could acknowledge several increasingly common HPE designs, including:

- mixed-methods studies
- longitudinal cohort studies
- adaptive educational trials
- Bayesian approaches
- non-inferiority educational studies
- equivalence testing
- multilevel mixed-effects educational research

Even if detailed formulas are beyond scope, acknowledging these designs and directing readers to appropriate resources would improve completeness.

**5. Greater emphasis on estimation rather than significance testing**

 Current methodological guidance increasingly recommends planning studies around estimation precision (confidence interval width) rather than solely statistical power.

 Although the manuscript discusses power extensively, confidence-interval-based planning receives relatively limited attention.

 A short section discussing estimation-based sample size planning would modernize the guide.

**Minor comments**

**6. Table 1 could be improved**

 Table 1 is very useful but could become an even stronger quick-reference resource.

 Consider adding columns for:

- primary statistical analysis
- commonly used software
- important assumptions
- common pitfalls

**7. Effect size section**

 The discussion regarding effect size is excellent.

 However, it would help readers if the manuscript also briefly discussed:

- minimally important educational differences
- educational versus statistical significance
- why published effect sizes should be interpreted cautiously because of publication bias.

**8. Survey studies**

 The survey section appropriately discusses prevalence estimation. However, many HPE surveys investigate associations or compare subgroups rather than estimating prevalence.

 The manuscript could briefly distinguish:

- estimation studies
- analytical cross-sectional studies
- psychometric surveys

since these require different sample size approaches.

**9. Instrument validation**

 The section would benefit from a stronger emphasis on current COSMIN recommendations.

 For example,

- factor loading strength
- communality
- model complexity

often influence required sample size more than simple participant-per-item ratios.

**10. Attrition**

 The attrition section is useful.

 It may also be helpful to mention:

- differential attrition
- missing data assumptions
- multiple imputation

because attrition affects more than simply recruitment targets.

**11. Reporting checklist**

 The reporting recommendations near the end are valuable.

 The manuscript could conclude with a concise reporting checklist or flowchart summarizing the required elements of a sample size justification.

 Such a figure would likely become one of the most frequently used parts of the paper.

**12. Figures**

 The manuscript currently contains tables but very few visual summaries.

 Consider adding:

- a decision tree for selecting sample size methods based on study design;
- a workflow diagram summarizing the nine-step process;
- a visual algorithm linking study design to appropriate formulas and software.

These additions would improve readability and educational value.

**Additional suggestions**

- Consider shortening repetitive explanations regarding effect size across multiple sections.
- Some paragraphs are relatively long and could be divided into shorter subsections for improved readability.
- The conclusion could include a brief discussion of future directions, such as AI-assisted sample size planning and simulation-based methods.

Is the rationale for developing the new method (or application) clearly explained?

Yes

Is the description of the method technically sound?

Partly

Are the conclusions about the method and its performance adequately supported by the findings presented in the article?

Yes

If any results are presented, are all the source data underlying the results available to ensure full reproducibility?

Yes

Are sufficient details provided to allow replication of the method development and its use by others?

Yes

Reviewer Expertise:

Medical education, Interprofessional education, Health professions education, Humanities, Bioethics

I confirm that I have read this submission and believe that I have an appropriate level of expertise to confirm that it is of an acceptable scientific standard, however I have significant reservations, as outlined above.

# Response

**Published:** 2026-08-14

**Authors:** Anusha Divvi

**Affiliations:** Sri Balaji Vidyapeeth University, Puducherry, Puducherry, India

We thank the reviewer for a detailed and constructive report.

**1. Clarify the methodology used to develop this guide.**

 A new section, *Methods: how this guide was developed*, has been added between Objectives and Main body. It states the three literature streams drawn on; the databases (MEDLINE/PubMed and Google Scholar), the search terms, and the use of forward and backward citation chaining; three explicit inclusion criteria; and an acknowledgement that no formal quality appraisal and no dual independent selection were performed. It closes by stating that the guide "has not been subjected to a Delphi or other formal consensus process, and it does not carry the authority of a consensus statement."

**2. Some recommendations are presented too prescriptively.**

 Accepted in full; this was the most consistent point across all three reports. It is addressed at four levels. The abstract Conclusions now state that "the numerical benchmarks reproduced here are illustrative anchors rather than methodological minima." Table 2 has been retitled *"Commonly cited sample size anchors for designs and methods in which a power-based calculation does not apply"*, its column heading changed from "Minimum or typical sample" to "Commonly cited anchor", and a standing caveat added beneath it. Each benchmark you named has been individually softened: the 12-per-group pilot rule is now "an order-of-magnitude anchor rather than a requirement"; the 30 for Cronbach's α is "commonly cited as providing reasonable precision" and should be "derived for the specific expected value rather than adopted as a fixed figure"; the 10:1 EFA rule is "the weakest of the available justifications"; and the 9–20 interview range now carries "these ranges describe what has been observed empirically in particular study contexts; they are not targets to be met." The new Limitations section states that applying these anchors outside their originating context "would repeat the error the guide is intended to correct."

**3. More discussion of software implementation.**

 A new section, *Software for sample size calculation*, has been added with new Table 3, mapping thirteen design types to G*Power 3.1 menu paths, R functions, and commercial equivalents (PASS, Stata, SAS, Mplus). All five packages you named are covered, and simr is included for simulation-based power. The section names the three errors we most often see in software-based calculation: selecting an independent-samples menu for paired data, entering a between-group *d* where the paired *d*z is required, and accepting a default one-sided test unintentionally and notes that software executes Step 7 only. Reporting the software, version and procedure is now item 13 of Table 4 and is written into Step 9.

**4. Limited discussion of complex contemporary designs.**

 A new section, *Designs and approaches beyond the scope of this guide*, covers all seven classes you listed: non-inferiority and equivalence; multilevel and mixed-effects; longitudinal cohort; adaptive designs; Bayesian approaches; and mixed methods, with the explicit statement that "a single sample size justification covering both strands is not appropriate." Each carries a signpost to the primary literature, and the section closes by stating that the guide is orientation rather than a substitute for specialist consultation.

**5. Greater emphasis on estimation rather than significance testing.**

 A new subsection, *Planning for precision rather than power*, has been added, with two worked examples and a new row in Table 1. It gives n = (Z₁₋α/₂ × σ/E)² for a single mean — 97 participants to estimate a mean examination score to within ±2 marks where σ = 10 — and n per group = 2Z₁₋α/₂²/E_d² for a standardised mean difference, yielding 193 per group to estimate an SMD to within ±0.20, "appreciably more than the 63 per group required merely to detect *d* = 0.5." Item 4 of the new checklist asks authors to declare whether the study is sized to detect or to estimate.

**6. Table 1 could be improved.**

 Table 1 has been expanded from four columns to six and from nine rows to twelve. *Primary analysis* is now column 3 and *Key assumption and common pitfall* column 6, with a standing assumptions note beneath the table. Software has been placed in a separate table rather than a fourth new column, because the G*Power path, R function and commercial equivalents cannot sit legibly in a Table 1 cell and Table 1 only works as a quick reference if it stays readable; Table 3 is cross-referenced from Table 1's parent section and from Step 7. Three rows have been added: analytical cross-sectional surveys, Delphi and consensus studies, and precision-based planning  so the table now covers every design discussed in the text.

**7. Effect size section.**

 A new subsection, *Educational importance, statistical significance, and the credibility of published effect sizes*, addresses all three points. On minimally important differences: there is no HPE-wide equivalent of the MID, but the half-standard-deviation finding is offered as a starting anchor, and authors are asked to state the difference they target and its basis. On educational versus statistical significance: "a statistically significant improvement of one percentage point on a summative examination in a large cohort may have no implication for curriculum design, while a non-significant difference of a full grade in a small cohort may be highly consequential and merely underpowered." On publication bias: "in a low-powered literature, the effects that reach significance are systematically the overestimates," with the recommendation to prefer a pooled estimate or, failing that, the lower bound of the reported confidence interval.

**8. Survey studies.**

 Restructured around the three purposes you distinguish, each with its own subheading. *Estimation surveys* retain the proportion formula and finite population correction. *Analytical cross-sectional surveys* are new, powered using the two-group formulas or the regression model actually to be fitted, with the note that a survey powered only for overall prevalence "will usually be underpowered for the comparisons the investigators intend to report." *Psychometric surveys* are directed to the validation section. A corresponding row has been added to Table 1 and a branch to Figure 2.

**9. Instrument validation.**

 Substantially rewritten. Requirements "depend far more on the strength of the factor loadings, the communalities of the measured variables, the number of factors extracted, and the ratio of variables to factors than on the number of participants per item." Where communalities are high and factors well determined, samples well below 10:1 can recover the structure; where communalities are low, 300 or more may be needed regardless of item count. COSMIN coverage has been strengthened, and the closing sentence asks authors to name the measurement property, state the precision or model-recovery requirement, and cite COSMIN "rather than a generic participant-per-item ratio."

**10. Attrition.**

 Retitled *Adjusting for expected attrition and missing data* and extended with three subsections, framed by "inflation protects statistical power; it does not protect validity." *Differential attrition* notes that "the resulting bias is not corrected by recruiting more participants." *Missing data mechanisms* distinguishes MCAR, MAR and MNAR. *Multiple imputation and analysis-stage implications* specifies what an imputation plan must state. The section has also been moved to precede the reporting section, correcting a forward reference in Version 1.

**11. Reporting checklist.**

 New Table 4, fifteen items in three groups: 1–10 for all designs, 11–13 covering inflation, missing data and software, and 14–15 covering non-power designs and the achieved sample. A prose reporting template is supplied in *Extended data*, File S1.

**12. Figures.**

 Two figures created; Version 1 had none. Figure 1 presents the nine-step workflow grouped into four labelled phases. Figure 2 is a decision tree routing the reader from study aim through the unit of analysis to the applicable method, with a common tail for attrition inflation and reporting and a flagged branch for designs needing specialist input. We combined the decision tree and design-to-formula algorithm deliberately, since presented separately they would have duplicated the same branching structure.

**Additional suggestions.** Effect-size discussion has been consolidated into Foundational principles, with Step 4 shortened to a cross-reference and the repetition in the two-group and SBE sections trimmed. Long paragraphs have been divided throughout. A new closing paragraph in Conclusions addresses both future directions you named: simulation-based design analysis, and the use of large language models in study design, which "can generate plausible-looking calculations without the design reasoning that makes a calculation defensible."
